# Spatio-temporal characterization of S- and M/L-cone degeneration in the Rd1 mouse model of retinitis pigmentosa

**DOI:** 10.1186/s12868-019-0528-2

**Published:** 2019-09-03

**Authors:** Daniel S. Narayan, Jack Ao, John P. M. Wood, Robert J. Casson, Glyn Chidlow

**Affiliations:** Ophthalmic Research Laboratories, Discipline of Ophthalmology and Visual Sciences, University of Adelaide, Level 7 Adelaide Health and Medical Sciences Building, North Terrace, Adelaide, SA 5000 Australia

**Keywords:** Rd1 mouse, Retinitis pigmentosa, Cone photoreceptor, Outer segment, Degeneration, Retina, S-opsin, M/L-opsin, Dual cone

## Abstract

**Background:**

The Pde6brd1 (Rd1) mouse is widely used as a murine model for human retinitis pigmentosa. Understanding the spatio-temporal patterns of cone degeneration is important for evaluating potential treatments. In the present study we performed a systematic characterization of the spatio-temporal patterns of S- and M/L-opsin^+^ cone outer segment and cell body degeneration in Rd1 mice, described the distribution and proportion of dual cones in Rd1 retinas, and examined the kinetics of microglial activation during the period of cone degeneration.

**Results:**

Outer segments of S- and M/L-cones degenerated far more rapidly than their somas. Loss of both S- and M/L-opsin^+^ outer segments was fundamentally complete by P21 in the central retina, and 90% complete by P45 in the peripheral retina. In comparison, degeneration of S- and M/L-opsin^+^ cell bodies proceeded at a slower rate. There was a marked hemispheric asymmetry in the rate of S-opsin^+^ and M/L-opsin^+^ cell body degeneration. M/L-opsin^+^ cones were more resilient to degeneration in the superior retina, whilst S-opsin^+^ cones were relatively preserved in the inferior retina. In addition, cone outer segment and cell body degeneration occurred far more rapidly in the central than the peripheral retina. At P14, the superior retina comprised a minority of genuine S-cones with a much greater complement of genuine M/L-opsin cones and dual cones, whilst the other three retinal quadrants had broadly similar numbers of genuine S-cones, genuine M/L-cones and dual cones. At P60, approximately 50% of surviving cones in the superior, nasal and temporal quadrants were dual cones. In contrast, the inferior peripheral retina at P60 contained almost exclusively genuine S-cones with a tiny minority of dual cones. Microglial number and activity were stimulated during rod breakdown, remained relatively high during cone outer segment degeneration and loss of cone somas in the central retina, and decreased thereafter in the period coincident with slow degeneration of cone cell bodies in the peripheral retina.

**Conclusion:**

The results of the present study provide valuable insights into cone degeneration in the Rd1 mouse, substantiating and extending conclusions drawn from earlier studies.

## Background

Retinitis pigmentosa (RP) comprises a diverse group of hereditary retinal dystrophies. Although the genetic mutations in RP are usually expressed in rods, in most phenotypes, secondary cone degeneration eventually ensues with loss of the remaining central vision [1]. Neuroprotection of cones is, therefore, a major therapeutic goal in RP.

In recent decades, a variety of animal models of RP have been developed. The Pde6brd1 (rd/rd, Rd1) mouse is a very widely used model of RP [1, 2], which is increasingly being employed to study the pathogenesis of secondary cone degeneration, and, to explore potential therapeutic interventions to delay this loss of cones. Rod degeneration in this strain occurs due to an autosomal recessive mutation in the beta subunit of rod photoreceptor cGMP-phosphodiesterase [3], the same enzyme defect identified in humans with autosomal recessive RP. As in the human disease, loss of rods is followed by a gradual, progressive loss of cones. The underlying pathogenesis of cone degeneration, both in the Rd1 strain and in RP, remains unclear, although a number of mechanisms have been implicated, including depletion of rod-derived cone viability factor, oxidative stress, microglial activation, and energetic failure [4–7].

To date, a number of studies—employing a variety of experimental methodologies—have shed light on certain aspects of cone degeneration in the Rd1 mouse [8–14]. Notable findings are that cone cell bodies and outer segments degenerate at markedly different rates, and, that there exists an asymmetry in cone degeneration across the retina. These findings have important implications for the design of neuroprotection studies, not least because markers of cone outer segments, such as the lectin peanut agglutinin, are frequently used to quantify cone survival.

Mice possess two types of cones that are distinguished by the portion of visible light spectrum to which they are sensitive. Short wavelength light stimulates S-cones, whilst medium to long wavelengths stimulates M/L-cones. These cones can be distinguished using well-characterised antibodies that specifically bind to the light-sensitive opsin protein in question. In wild-type (WT) mouse retinas, S- and M/L-opsins are expressed in the outer segments of S- and L/M-cones, respectively [15], while in Rd1 mice, cone opsins are redistributed to the soma upon outer segment degeneration [8, 16, 17]. There are also a number of cones that express both S- and M/L-opsins in their outer segments, which are referred to as dual cones [18]. While detailed information is available on the spatial distribution of genuine S-opsin cones, genuine M/L-opsin cones, and dual cones in the retinas of albino and pigmented wild-type mouse strains [15], as well as albino and pigmented wild-type rat strains [19], hitherto, no data are available concerning the distribution and proportion of dual cones in the degenerating Rd1 retina.

The aims of the present study were to perform, for the first time, a systematic characterization of the spatio-temporal patterns of S- and M/L-opsin^+^ cone outer segment and cell body degeneration in Rd1 mice, to describe the distribution and proportion of dual cones in Rd1 retinas, and to examine the kinetics of microglial activation during the period of cone degeneration in the Rd1 retina. The combined data will provide a valuable baseline for future studies investigating cone neuroprotection in the Rd1 mouse.

## Methods

### Animals and procedures

This study was approved by the Animal Ethics Committees of SA Pathology/Central Adelaide Local Health Network and the University of Adelaide (Adelaide, Australia) and conformed with the Australian Code of Practice for the Care and Use of Animals for Scientific Purposes, 2013, and with the ARVO Statement for the use of animals in vision and ophthalmic research. C3H/HeJArc (Pde6brd1; Rd1) mice and C57BL/6J mice were obtained from Animal Resources Centre (Perth, Australia). The Pde6brd1 mouse is an inbred strain homozygous for the PDE6b mutation. Rd1 mice were inbred to produce offspring. All animals were housed in a temperature and humidity controlled room with a 12-h light and 12-h dark cycle, and provided with food and water and environmental enrichment ad libitum. Rd1 mice were humanely killed at various postnatal day (P) time points for detection of cone opsin proteins by immunohistochemistry and for detection of mRNA expression of cone opsins. For the spatio-temporal characterisation of cone degeneration by immunohistochemistry on wholemounts, the number of samples analysed was as follows: P14 (n = 7), P21 (n = 5), P30 (n = 7), P45 (n = 7), P60 (n = 7), P75 (n = 4), P90 (n = 6), P150 (n = 5) and P300 (n = 5). Six WT (C57BL/6) mice were humanely killed at P60. For determination of the proportion of genuine S-cones, genuine M/L-cones and dual cones, the number of samples analysed was as follows: P14 (n = 5), P60 (n = 24). For real-time RT-PCR, the number of samples analysed was as follows: WT (n = 6), P14 (n = 6), P30 (n = 6), P45 (n = 9), P60 (n = 15), P90 (n = 6). For the characterisation of microglia by immunohistochemistry on wholemounts and transverse sections, the number of samples analysed was as follows: transverse sections: P7, P14, P21, P30, P45, P60, P75 (all n = 3); wholemounts P21 (n = 4), P45 (n = 6), P60 (n = 6), P300 (n = 4).

### Tissue processing and immunohistochemistry

All mice were euthanized by transcardial perfusion with physiological saline under terminal anaesthesia (100 mg/kg body weight ketamine and 10 mg/kg body weight xylazine) followed by decapitation. The superior aspect of each cornea was marked before globes were enucleated. For wholemount immunohistochemistry, eyes were fixed in 4% (w/v) neutral buffered formalin for 24 h and dissected into posterior eye-cups. The corneal mark was used to orient the eye and a small radial cut was made in the superior retina while it was still attached to the retinal pigment epithelium (RPE) in the eye-cup. Retinas were removed and prepared as flattened wholemounts by making another four radial cuts. Retinal wholemounts were then stored in phosphate buffered saline (PBS). For double labeling immunohistochemistry, retinas were incubated in PBS containing 1% (v/v) Triton X-100 detergent (T) for 1 h at room temperature. Next, retinas were incubated in PBS-T containing 3% (v/v) normal horse serum (NHS-T) for 1 h at room temperature to block non-specific antibody binding. Retinas were then incubated overnight at 4 °C with a combination of primary antibodies diluted in NHS-T. OPN1SW antibody was used to detect short-wave sensitive cones, whilst anti-R/G opsin antibody was used to detect medium/long wave sensitive opsin cones (see Table 1). On day 2, retinas were washed for 1 h at room temperature in PBS-T, then incubated overnight at 4 °C with a combination of AlexaFluor-488 and -594 conjugated secondary antibodies (1:250; Invitrogen, Carlsbad, CA) diluted in NHS-T. Finally, retinas were washed in PBS for 1 h at room temperature prior to mounting with the photoreceptor side facing up, using anti-fade mounting medium (Dako, California, USA).

**Table 1:** Primary antibodies used in the study

Protein	Source	Clone/cat. no.	Species	Immunogen	Dilution
CD68	Bio-Rad	clone FA-11	Rat	Purified concanavalin A acceptor glycoprotein from P815 cell line	1:500^a^
Iba1	Wako	019-19741	Rabbit	Synthetic peptide corresponding to C-terminus of Iba1	1:4000^a^ 1:20,000
M/L-opsin	Merck-Millipore	AB5405	Rabbit	Recombinant human red/green opsin	1:1500^a^ 1:5000
Rhodopsin	Abcam	clone RET-P1	Abcam	Membrane preparation from adult rat retina	1:1000
S-opsin	Santa-Cruz	sc-14363	Goat	Peptide mapping at the N-terminus of the opsin protein encoded by OPN1SW of human origin	1:1500^a^

^a^Dilution used for 2-step fluorescent immunostaining procedure. Wako Pure Chemicals, Osaka, Japan; Bio-Rad, Gladesville, Australia; Santa Cruz Biotechnology, CA, USA; Merck Millipore, Kilsyth, Australia; Abcam, Cambridge, UK; Dako, Carpintería, CA, USA

For immunohistochemistry on transverse sections, globes were immersion-fixed in Davidson’s solution for 24 h and transferred to 70% ethanol until processing. Davidson’s solution, which comprises 2 parts formaldehyde (37%), 3 parts 100% ethanol, 1 part glacial acetic acid and 3 parts water, is the preferred fixative for whole eyes as it provides optimal tissue morphology while avoiding retinal detachment. Globes were embedded sagittally and 4 μm serial sections were cut. Colorimetric immunohistochemistry was performed as previously described [20, 21]. In brief, tissue sections were deparaffinised, endogenous peroxidase activity was blocked, and high-temperature antigen retrieval was performed. Subsequently, sections were incubated in primary antibody (Table 1), followed by consecutive incubations with biotinylated secondary antibody and streptavidin-peroxidase conjugate. Color development was achieved using NovaRED substrate kit (Vector Laboratories Inc., Burlingame, CA, USA).

For double-labeling immunofluorescence of transverse retinal sections, samples were treated exactly as described above except that a combination of primary antibodies was used. Detection of signal was achieved by incubation with the appropriate combination of AlexaFluor-488 and -594 conjugated secondary antibodies (1:250; Invitrogen, Carlsbad, CA) instead of streptavidin–peroxidase conjugate. Sections were mounted using anti-fade mounting medium.

### Image acquisition and quantification

Confirmation of the specificity of antibody labeling was judged by the morphology and distribution of the labeled cells, by the absence of signal when the primary antibody was replaced by isotype/serum controls, and, by comparison with the expected staining pattern based on our own, and other, previously published results. Retinal wholemounts and transverse sections were examined under a fluorescence microscope (BX-61; Olympus, Mount Waverly, Victoria, Australia) equipped with a scientific grade, cooled CCD camera. Wholemount images were taken using a Hamamatsu NanoZoomer 2.0-HT fluorescence module and viewed using the Hamamatsu NanoZoomer Digital Pathology system. Photomicrographs were captured using an excitation wavelength of 488 nm to detect Alexafluor 488-labeled antibodies and an excitation wavelength of 594 nm to detect Alexafluor 594-labeled antibodies. Transverse retinal sections were photographed using a light microscope.

For quantification of the spatio-temporal characterisation of cone degeneration by immunohistochemistry on wholemounts, four photomicrographs of the central retina (measuring 720 μm × 540 μm) were taken directly superior, temporal, inferior, and nasal to the centre of the optic disc, and, four photomicrographs of the peripheral retina (measuring 720 μm × 540 μm) were taken 1.5 mm superior, temporal, inferior, and nasal from the centre of the optic disc, yielding a total of 8 images per fluorescent channel per retina. For determination of the proportion of genuine S-cones, genuine M/L-cones and dual cones, four photomicrographs of the peripheral retina (measuring 526.5 × 422.5 µm), one in each of the retinal quadrants, were photographed 1.5 mm from the optic disc. Quantification of cone survival was performed using Image-J software (NIH, Bethesda, Maryland, USA). Initially, however, images were processed in Photoshop CS3 (Adobe, San Jose, California, USA) for uneven lighting (using a flatten filter), sharpened, levels enhanced, and finally converted to 8-bit mode. Cone cell bodies were identified by boththeir wide ovoid morphology, segments by their narrow, bundle-like appearance. Outer segments label for cone opsins with high fluorescent intensity. In comparison, cell bodies label with lower fluorescent intensity. These different cellular structures can therefore be analysed and quantified separately. The area in pixels was calculated with the “analyse particles” function. Essentially, the same method was used to quantify Iba1 and CD68 labeled microglia. To quantify the number of Iba1-positive cells in transverse sections of the retina, four images were captured across each immunolabeled retina, a process which was independently repeated for a second labeled section.

For characterisation of the proportion of genuine M/L-cones, genuine S-cones, and dual cones in P14 and P60 Rd1 mice, initial image processing was performed using Photoshop CS3. Each pair of S-opsin and M/L-opsin images were corrected for uneven lighting using a flatten filter, and where necessary the linear gradient tool, and subsequently adjusted to be of approximately the same labeling intensity. The images were then merged (M/L-opsin, green channel; S-opsin, red channel), sharpened, levels enhanced, filtered for “noise/dust” and finally converted to 8-bit mode. In Image-J software, colour thresholding was performed on each merged image to determine the proportion of “red”, “yellow” and “green” cones, which correspond to “genuine S-cones”, “dual cones”, and “genuine M/L-cones”, respectively. To facilitate this goal, the total area of “all cones” within the image was first determined by manually thresholding using the “brightness” scale, with “saturation” and “Hue” set at the maximum range of 0–255. Next, to delineate the proportion of individual cone types, the “Hue” scale was altered to individually detect red, yellow, and green colours. For each “Hue” setting, the area in pixels was calculated using the “analyze particles” function, and a mask of the labeled area was produced.

### Real-time RT-PCR

All mice were killed by transcardial perfusion with physiological saline under deep anaesthesia. Retinas were carefully dissected, total RNA was isolated, and first strand cDNA was synthesized from DNase-treated RNA samples. Real-time PCR reactions were then carried out in 96-well optical reaction plates using the cDNA equivalent of 10 ng total RNA for each sample in a total volume of 20 μl containing 1× SYBR Green PCR master mix (BioRad) forward and reverse primers. Thermal cycling conditions were 95 °C for 3 min and 40 cycles of amplification comprising 95 °C for 12 s, 63 °C for 30 s and 72 °C for 30 s. Primer pairs (Table 2) were designed from sequences contained in the GenBank database using the primer design software Primer 3 (http://bioinfo.ut.ee/primer3-0.4.0/primer3/) and were selected wherever possible to amplify sequences that spanned at least one intron. Primer sequences were analyzed for *T*_*m*_ (melting temperature), secondary structure and primer-dimer formation with NetPrimer analysis software (http://www.premierbiosoft.com/netprimer). PCR assays were performed using the CFX cycler (Bio-Rad) and all samples were run in duplicate. After the final cycle of the PCR, primer specificity was checked by the dissociation (melting) curve method and by electrophoresis of PCR products on 3% agarose gels. All mRNAs were amplified with high efficiency and linearity during real-time PCR. Mean amplification efficiencies, as determined by plotting cycle threshold as a function of initial cDNA quantity, were in the range of 1.90–2.00. Results obtained from the real-time PCR experiments were, therefore, quantified using the comparative threshold cycle (C_T_) method (ΔΔC_T_) for relative quantitation of gene expression, with a minor correction for amplification efficiency. All values were normalised to a pool of two different endogenous reference genes, GAPDH and cyclophilin, and expressed relative to WT retinas and relative to the earliest time point analysed, P14. Statistical analysis was carried out by one-way ANOVA followed by post hoc Dunnett’s Multiple Comparison Test.

**Table 2:** Primer sequences for mRNAs amplified by real-time RT-PCR

mRNA	Primer sequences	Product size (bp)	Accession number
GAPDH	5′-TGCACCACCAACTGCTTAGC-3′ 5′-GGCATGGACTGTGGTCATGAG-3′	87	NM_001289726
Cyclophilin	5′-GTGTTCTTCGACATCACGGCT-3′ 5′-CTGTCTTTGGAACTTTGTCTGCA-3′	82	NM_008907
S-opsin	5′-CCTCTTTCCCTCATCTGCTTCTC-3′ 5′-ACCTCCCGTTCAGCCTTTTG-3′	107	NM_007538
M/L-opsin	5′-CATCCGAGCAGTGGCAAAG-3′ 5′-CACAAGAGGGTGGAAGGCATAG-3′	175	NM_008106

## Results

### Identification of S- and M/L-cones in wild-type and Rd1 retinas

S- and M/L-cones were identified by immunodetection using well-characterised antibodies [15, 19]. In WT retinas, each opsin was principally associated with the outer segment (Additional file 1). Lower intensity labeling of the cone somata and synaptic terminal was also detectable, which was stronger in S-opsin^+^ cones than in M/L opsin^+^ cones. Previous studies have demonstrated that in Rd1 mice, cone outer segments degenerate prior to death of the cell body, resulting in ectopic redistribution of opsins to the surviving cell body [8]. Thus, in Rd1 retinas, cones with intact outer segments, as well as cones devoid of outer segments, are both detectable. In the present study, cone cell bodies were identified by their wide ovoid morphology and labeled with moderate fluorescent intensity, whilst outer segments were identified by their narrow bundle-like appearance and labeled with high fluorescent intensity (Additional file 2). These different cellular structures were analysed and quantified separately using image thresholding (Additional file 2). Ortin-Martinez et al. [15] noted that reliable quantification of cone segments in wholemounts depends upon meticulous removal of the RPE without damaging the delicate outer segments. Retinas used in this study were successfully dissected from the RPE, however, it is possible that this was not always the case. Unlike a healthy retina from a wild-type mouse, Rd1 retinas display non-homogeneous cone degeneration. It is therefore possible that any apparent heterogeneous degeneration may be partly artefactual in nature.

### Spatio-temporal characterisation of S- and M/L-cone degeneration in Rd1 retinas

Prior to examination of cone degeneration, we verified the timing of rod photoreceptor degeneration in our cohort of Rd1 mice by immunolabelling for rhodopsin using the well-charactersied antibody clone RET-P1. The results showed that rod death is an early phenotypic event. By P21, only a very sparse population of rods were still evident (Additional file 3), which had been completely lost by P28. The results are in agreement with the large volume of research conducted on Rd1 mice [2].

**Fig. 1:**
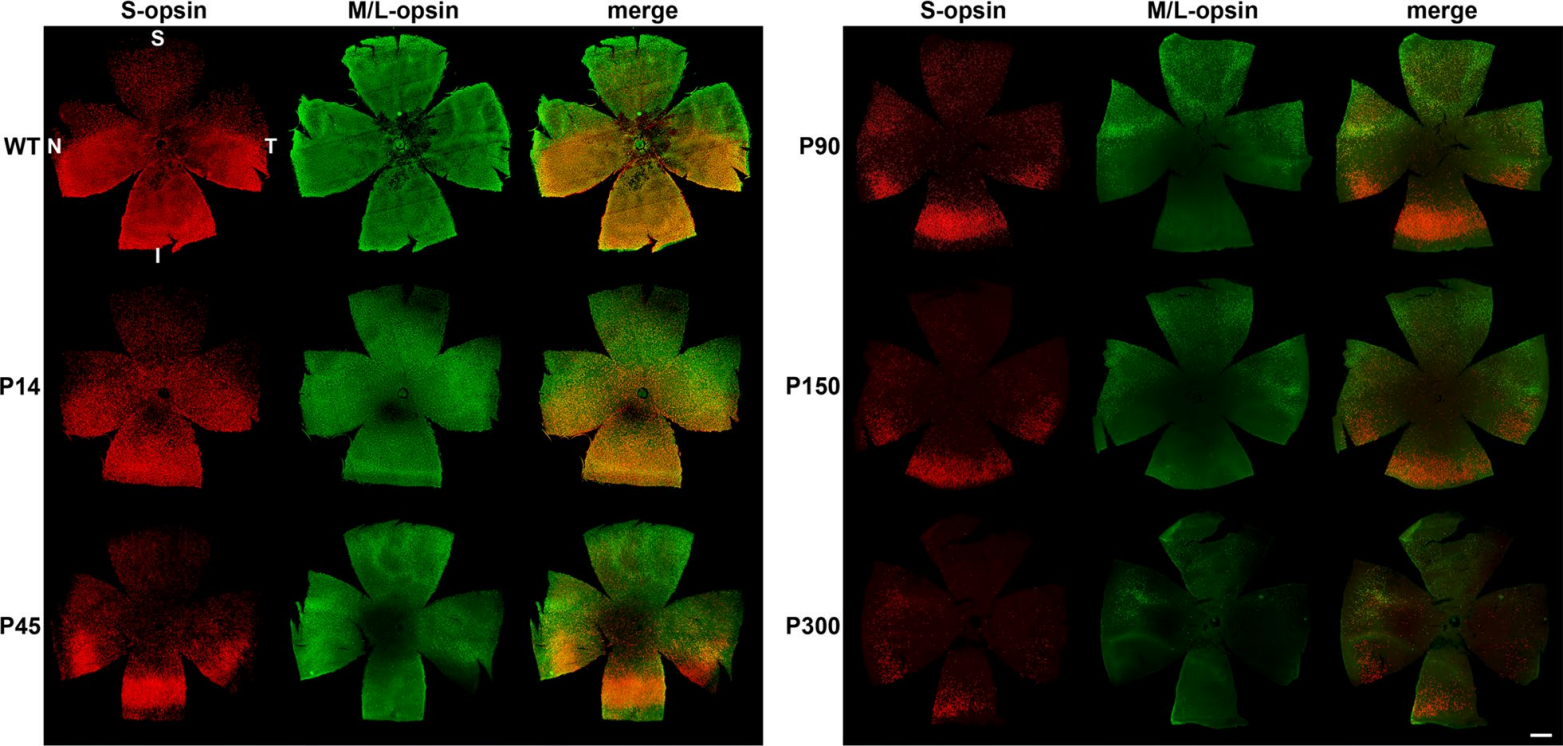
Representative, low magnification, images of S-opsin^+^ cones and M/L-opsin^+^ cones in retinal wholemounts of C57BL/6 wild-type (WT) and of Rd1 mice from postnatal day (P) 14 to P300. Double labeling immunofluorescence was performed using antibodies directed against S-opsin (red) and M/L-opsin (green). Cell bodies and outer segments cannot be readily differentiated in these low magnification images; however, the topographical pattern of cone degeneration is apparent. *S* superior, *I* inferior, *N* nasal, *T* temporal. Scale bar: 500 μm

**Fig. 2:**
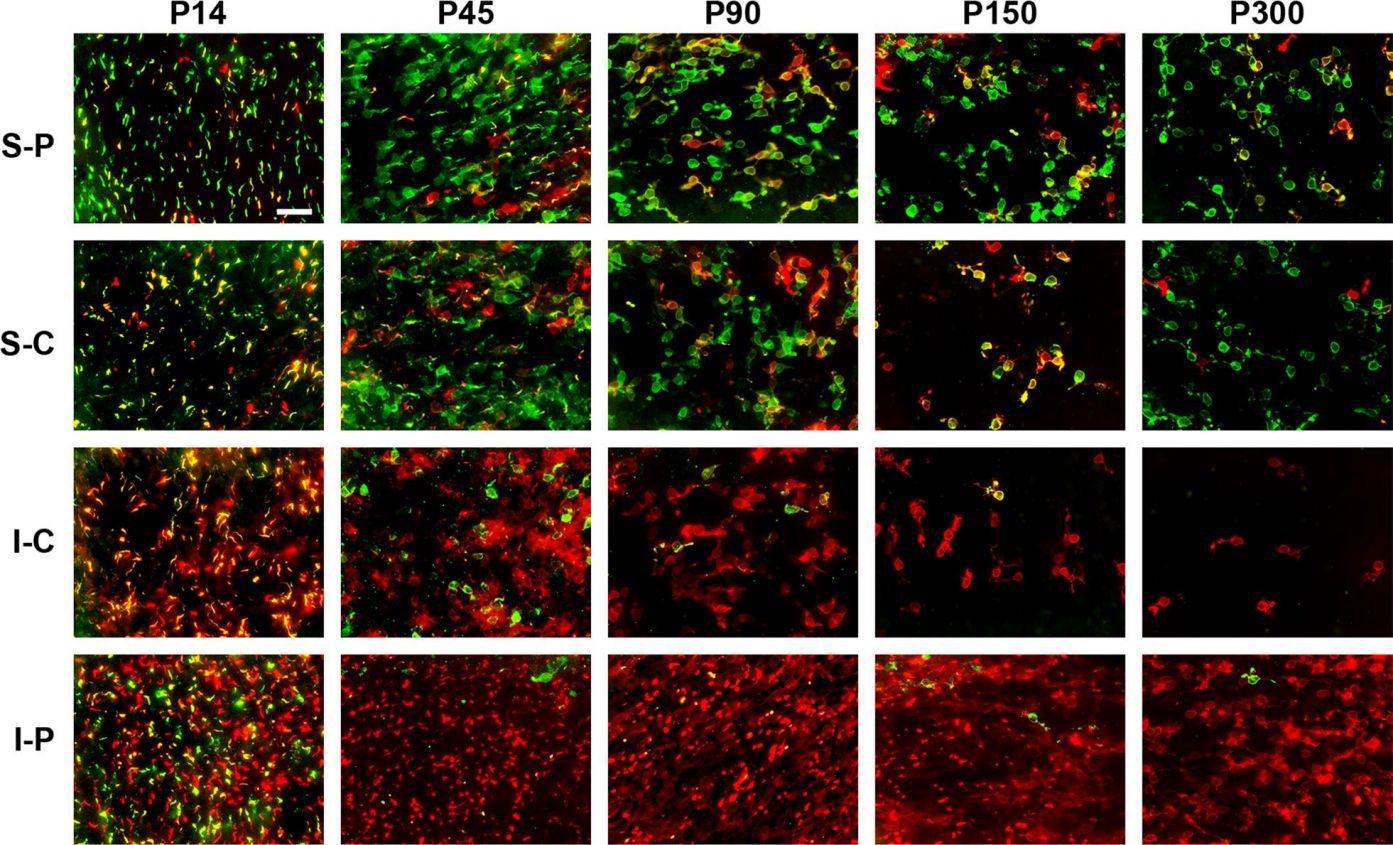
Representative, high magnification, images of S-opsin^+^ cones and M/L-opsin^+^ cones in retinal wholemounts of Rd1 mice from postnatal day (P) 14 to P300. Double labeling immunofluorescence was performed using antibodies directed against S-opsin (red) and M/L-opsin (green). Outer segments of both cone types label intensely at P14. By P45, large numbers of outer segments had degenerated, allowing better visualisation of cone cell bodies. *S-P* superior-peripheral retina, *S-C* superior-central retina, *I-C* inferior-central retina, *I-P* inferior-peripheral retina. Scale bar: 100 μm

**Fig. 3:**
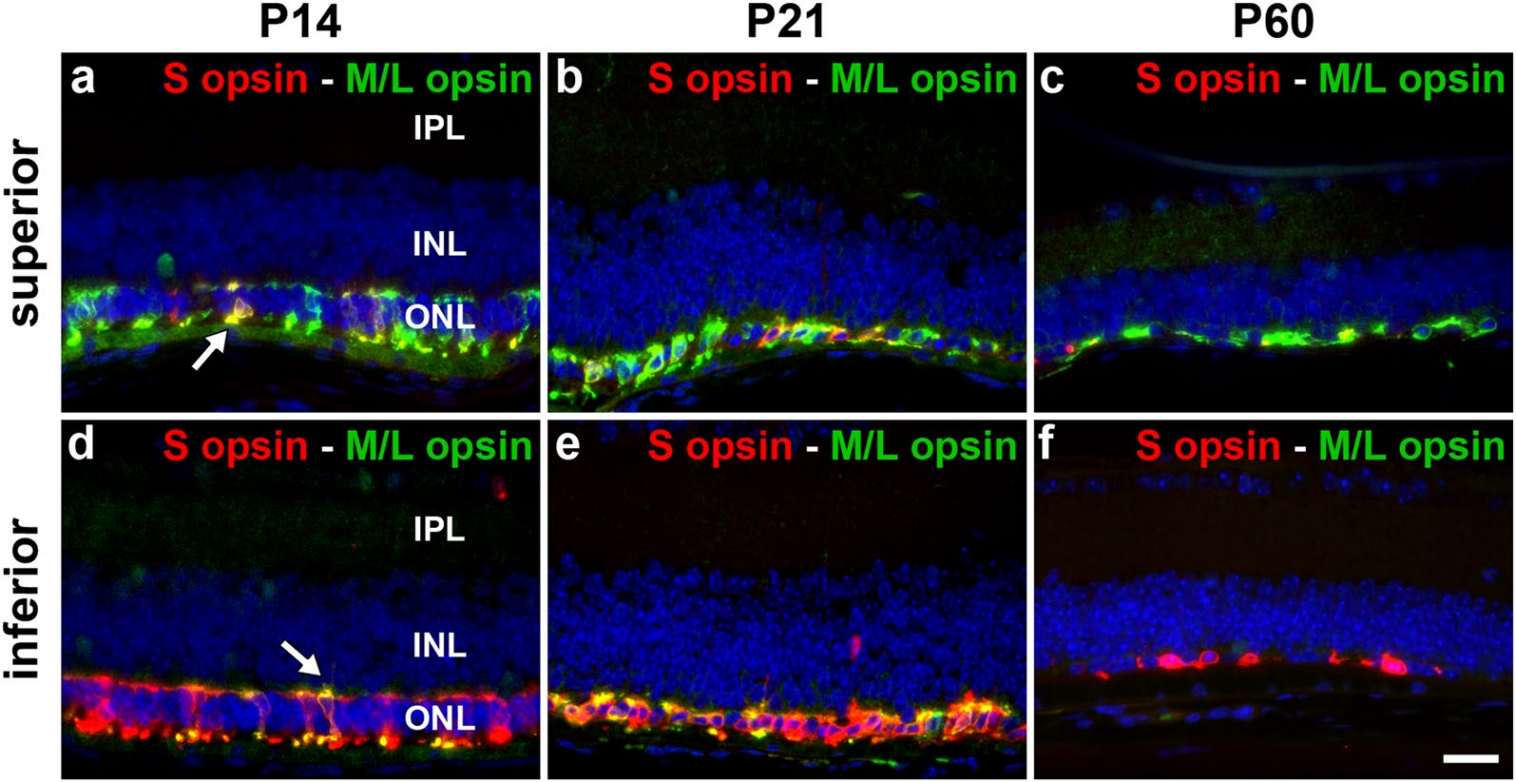
Representative images of S-opsin^+^ cones and M/L-opsin^+^ cones in retinal transverse sections of Rd1 mice at postnatal day (P) 14, P21 and P60. Double labeling immunofluorescence in the superior (**a**–**c**) and inferior retina (**d**–**f**) was performed using antibodies directed against S-opsin (red) and M/L-opsin (green). Dual cones express both S-opsin and M/L-opsin and appear yellow (arrows). Scale bar: 20 mm. *IPL*, inner plexiform layer; *INL*, inner nuclear layer; *ONL*, outer nuclear layer

Our results showed a homogeneous distribution of M/L-opsin^+^ cones throughout the Rd1 retina at P14, whilst there was a gradient of S-opsin^+^ cones, from a relatively sparse population in the superior retina to a high density of cones in the inferior retina (Figs. 1, 2, 3). These results are in agreement with previous studies that examined the distribution of cones in the Rd1 retina at a very early stage of degeneration [8, 9]. At P14, the earliest time point analysed, the vast majority of S-opsin^+^ and M/L-opsin^+^ cones had preserved outer segments, but the segments were typically misshapen or swollen in appearance, whilst ectopic redistribution of opsins to the surviving cell bodies had commenced to a variable degree (Fig. 3; Additional files 4, 5). The high concentration of intensely-labeled outer segments at P14 made cone cell body identification in wholemounts problematic. By P21, a significant proportion of outer segments had degenerated (Fig. 3; Additional files 4, 5). Therefore, cone cell body density was only measured from P21.

**Fig. 4:**
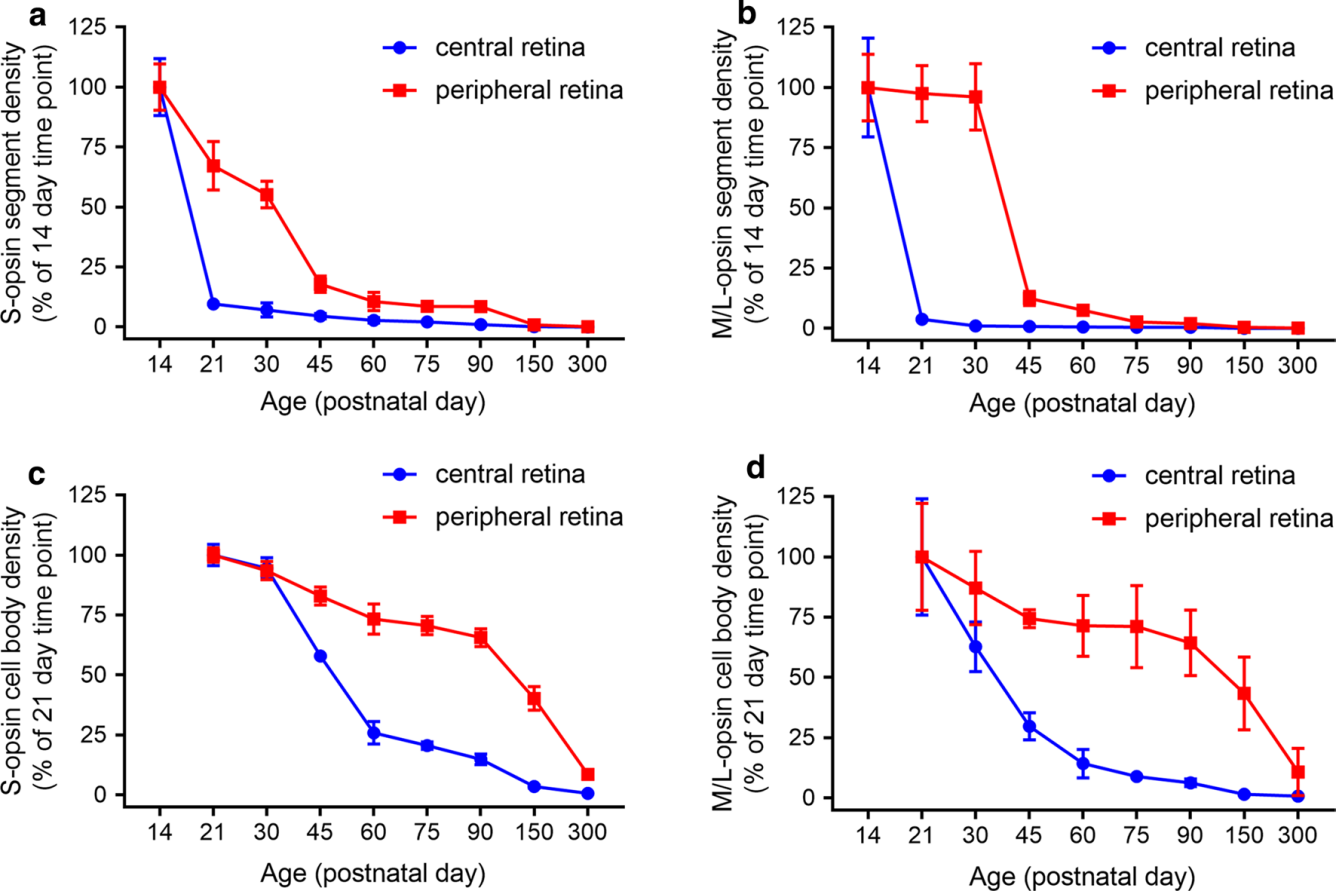
Graphs showing the natural his tory of S-opsin^+^ and M/L-opsin^+^ cone degeneration in Rd1 mice over time. **a**, **b** Quantification of S-opsin^+^ and M/L-opsin^+^ cone outer segment degeneration in the central and peripheral retina. **c**, **d** Quantification of S-opsin^+^ and M/L-opsin^+^ cone cell body degeneration in the central and peripheral retina. Data represent mean ± SEM

**Fig. 5:**
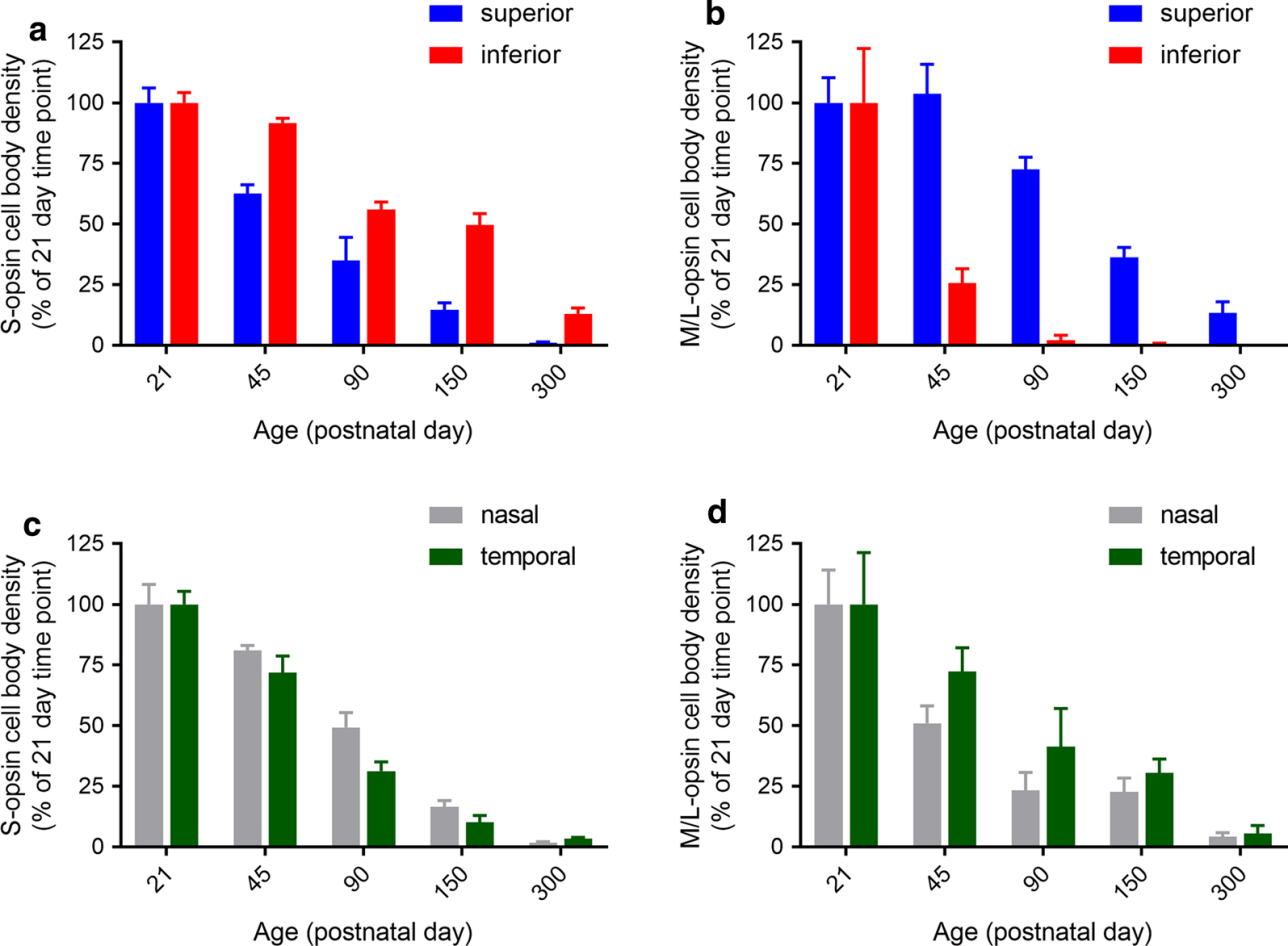
Spatio-temporal quantification of S-opsin^+^ and M/L-opsin^+^ cone cell body degeneration in the superior and inferior (**a**, **b**), and, nasal and temporal (**c**, **d**) peripheral retinal quadrants. Data represent mean ± SEM

Quantification of the temporal progression of S-opsin^+^ and M/L-opsin^+^ cone degeneration in wholemounts revealed a striking difference between central and peripheral areas of the retina, and, between outer segments and cell bodies. For both S-opsin^+^ and M/L-opsin^+^ cells, cone outer segment degeneration in the central retina was effectively complete by P21 (Figs. 2, 4a, b). In contrast, outer segment degeneration in the peripheral retina was more gradual, albeit that greater than 90% of S-opsin^+^ and M/L-opsin^+^ outer segments had still degenerated by P45 (Figs. 2, 4a, b). Prior to P30, M/L-opsin^+^ segments in the peripheral retina appeared somewhat more preserved than S-opsin^+^ segments (Fig. 4a, b). With regard to cell bodies, there was likewise a more rapid degeneration of both S-opsin^+^ and M/L-opsin^+^ cones in the central retina than in the peripheral retina (Figs. 2, 4c, d). For example, relative to P21, there was only 25% survival of S-opsin^+^ cones in the central retina, but approximately 75% remaining in the peripheral retina (Fig. 4c). The overall kinetics of S-opsin^+^ and M/L-opsin^+^ cone cell body loss in the central and peripheral retina were similar (Fig. 4c, d), but there was a marked disparity in the spatial pattern of degeneration of each cone type. There was hemispheric asymmetry in the rate of S-opsin^+^ and M/L-opsin^+^ cone degeneration; thus, S-opsin^+^ cones were remarkably well preserved in the inferior retina relative to the superior retina (Figs. 1, 2, 3, 5a), whilst M/L-opsin^+^ cones were much more resilient to degeneration in the superior relative to the inferior retina (Figs. 1, 2, 3, 5b). The disparity became more pronounced as cone degeneration progressed (Figs. 1, 2, 3, 5a, b). By P300, small numbers of M/L-opsin^+^ cones survived in the superior peripheral retina, while a small population of S-opsin^+^ cones remained in the peripheral inferior retina (Figs. 1, 2, 5a, b). Loss of S-opsin^+^ and M/L-opsin^+^ cone cell bodies in the nasal and temporal quadrants of the retina displayed similar kinetics to each other (Fig. 5c, d).

**Fig. 6:**
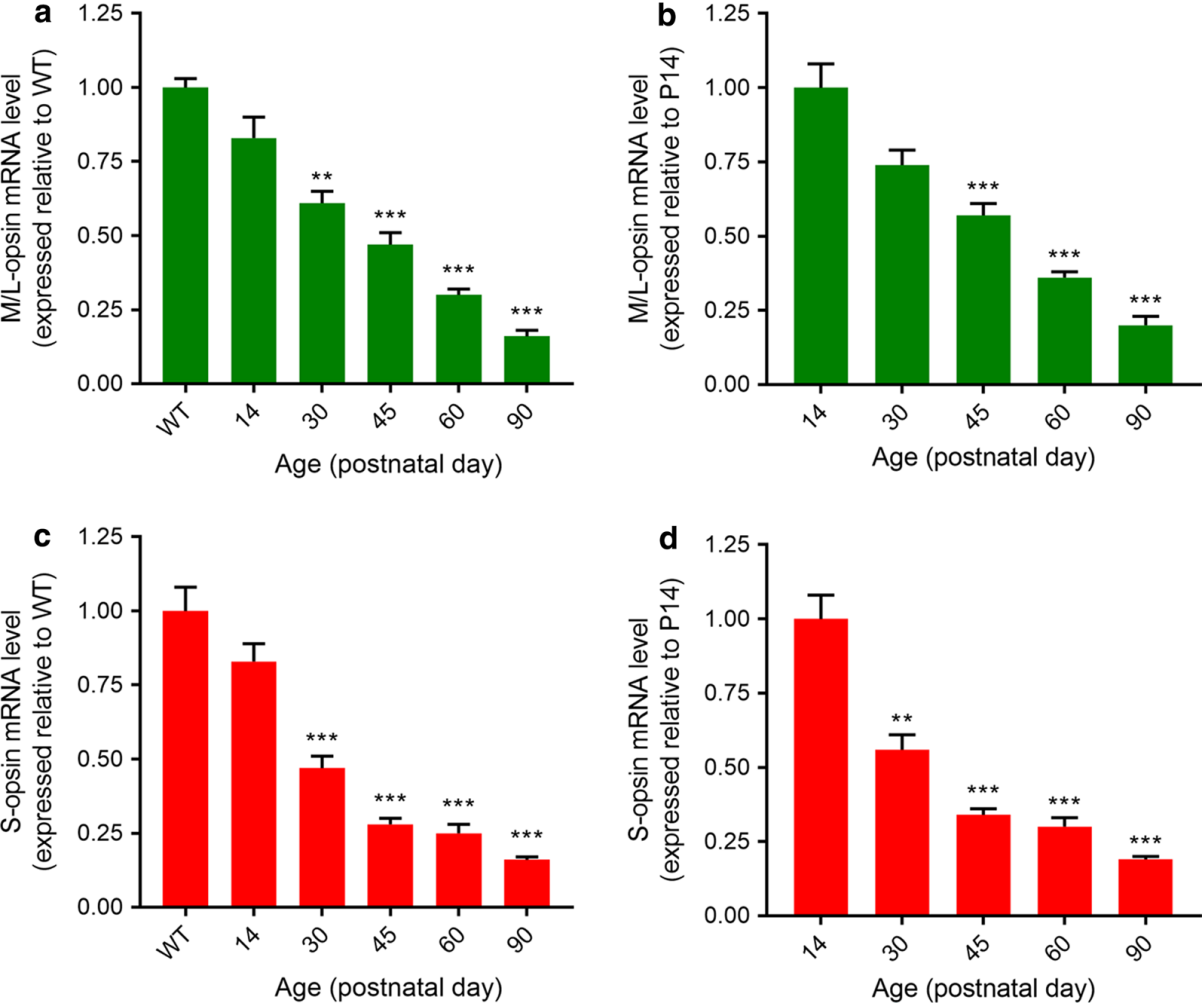
Quantification of M/L-opsin (**a**, **b**) and S-opsin (**c**, **d**) mRNA levels in Rd1 retinas from postnatal day (P) 14 to P90. Values (represented as mean ± SEM) are normalised to a pool of two endogenous genes (GAPDH and cyclophilin) and expressed relative to WT retinas (**a**, **c**) and to the 14d Rd1 time point (**b**, **d**). **P < 0.01, ***P < 0.001, by ANOVA followed by post hoc Dunnett’s Multiple comparison test (**a**, **c** vs WT; **b**, **d** vs 14 days Rd1)

To impart perspective on the opsin protein results, we utilised qPCR to examine the levels of M/L- and S-opsin mRNAs in Rd1 retinas (normalised to a pool of two reference genes). Relative to WT retinas, the levels of both S-opsin and M/L-opsin mRNAs were lower by P14, albeit the differences did not reach significance at this time (P = 0.43; P = 0.56, respectively; Fig. 6). Thereafter, the level of M/L-opsin mRNA decreased in a linear fashion from P14 until P90, at which point it was 16% of the WT level (Fig. 6a). The amount of S-opsin mRNA decreased marginally more rapidly than M/L-opsin mRNA, but had also been reduced to 16% of the WT level by P90 (Fig. 6c). Relative to P14, the decrease in M/L-opsin mRNA was not statistically significant at P30 (P = 0.09; Fig. 6b), but the level of S-opsin mRNA was significantly lower at the same time point (P < 0.05; Fig. 6d). The decreases in both cone opsin mRNAs were statistically significant from P45 onwards. Overall, the qPCR data were concordant with those obtained from immunohistochemistry of wholemounts and transverse sections.

### Investigation of dual cones in Rd1 retinas

The second part of this study involved characterizing the population of genuine S-cones, genuine M/L-cones and dual cones in the Rd1 mouse retina at the onset of cone degeneration, P14, and at the approximate midpoint of degeneration, P60. These data are available in the retinas of WT mouse strains [15], but not in the Rd1 retina. Retinal wholemounts were immunolabeled with antibodies to S- and M/L-opsins. Images from the peripheral retina were captured, pre-processed, merged, and the cone densities of genuine S-opsin cones, genuine M/L-opsin cones and dual cones then quantified in each of the four retinal quadrants using colour thresholding (see “Methods”). At P14, outer segments were quantified, while at P60 cell bodies (plus any surviving outer segments) were used. Initially, however, we tested the methodology using a retinal wholemount from a WT mouse (see Additional files 6 and 7). Examination of this retina revealed 14% genuine S-cones, 35% dual cones and 50% genuine M/L-cones, values that are not dissimilar to those previously reported [15].

**Fig. 7:**
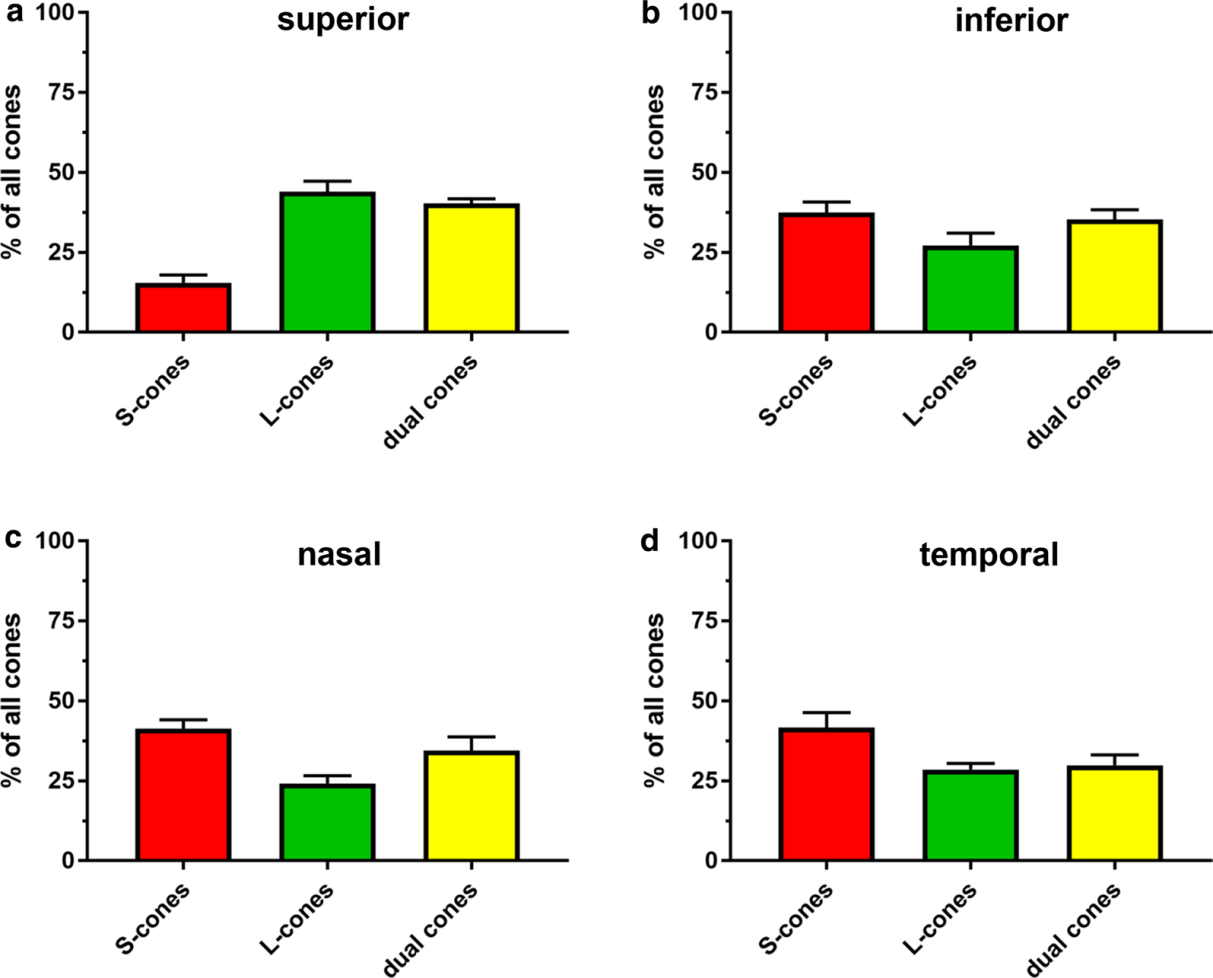
Bar graphs displaying composition of the cone population in the superior (**a**), inferior (**b**), nasal (**c**) and temporal (**d**) quadrants of the Rd1 retina at P14. Data represent mean ± SEM

At P14, the superior retina (Fig. 7, Additional file 8) comprised a minority of genuine S-cones (15.6%), as expected, with a much higher complement of genuine M/L-opsin cones (38.1%) and dual cones (40.4%). In contrast, the inferior retina (Fig. 7, Additional file 9) contained relatively similar numbers of genuine S-cones (37.5%), genuine M/L-cones (27.2%) and dual cones (35.3%). Nasal (Fig. 7, Additional file 10) and temporal (Fig. 7, Additional file 11) regions had similar numbers of genuine S-cones (41.3%; 41.6%, respectively), genuine M/L-opsin cones (24.2%; 28.5%, respectively) and dual cones (34.5%; 29.9%, respectively).

**Fig. 8:**
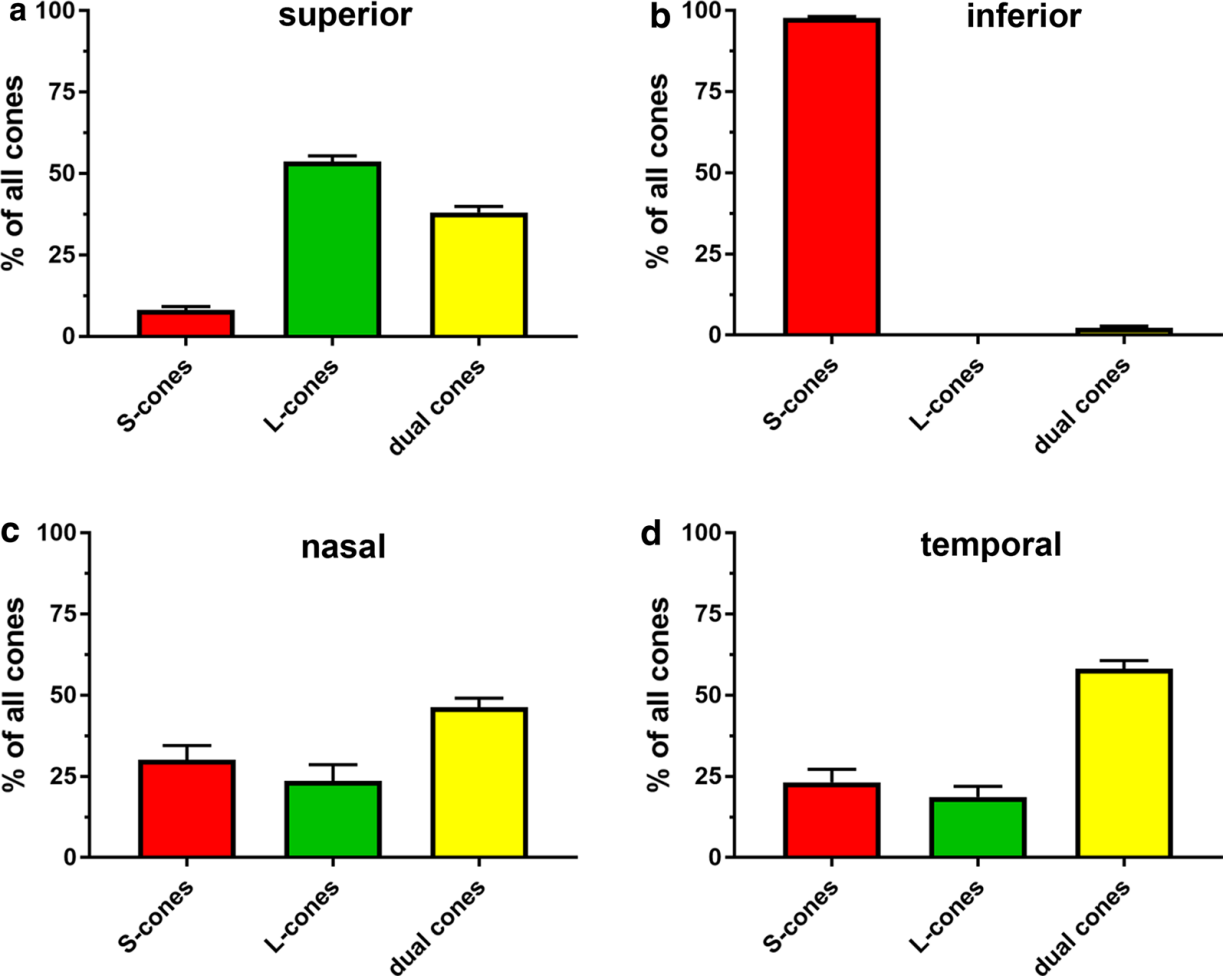
Bar graphs displaying composition of the cone population in the superior (**a**), inferior (**b**), nasal (**c**) and temporal (**d**) quadrants of the Rd1 retina at P60. Data represent mean ± SEM

In agreement with the results from the spatio-temporal characterisation of S- and M/L-cone degeneration, there was a marked divergence in cone composition between the superior (Fig. 8, Additional file 12) and inferior (Fig. 8, Additional file 13) retina at P60. The superior retina at P60 consisted of a majority of dual cones (53.7%), followed by genuine M/L-cones (38.1%) and a sparse population of genuine S-cones (8.2%). In contrast, the inferior retina at P60 contained almost exclusively genuine S-cones (97.7%) with a small minority of dual cones (2.3%), and no identifiable genuine M/L-cones. Nasal (Fig. 8, Additional file 14) and temporal (Fig. 8, Additional file 15) regions had similar compositions of genuine S-cones (23.2%; 30.2%, respectively), genuine M/L-cones (18.6%; 23.7%, respectively) and dual cones (46.3%; 58.1%, respectively). In terms of overall cone survival in the four quadrants at P60, i.e. combined presence of all cone types, the results showed approximately threefold greater overall cone survival in the inferior retina (1,154,292 ± 42, 610 pixels^2^) compared with the superior (342,175 ± 30,593 pixels^2^), nasal (402,722 ± 59,855 pixels^2^), and temporal (390,130 ± 52,139 pixels^2^) quadrants. This results exemplifies the remarkable survival of S-opsin^+^ cones in the peripheral regions of the inferior retina (see Fig. 8, Additional file 13).

### Spatio-temporal characterisation of microglial responses in Rd1 retinas

In the third part of this study we investigated microglial/macrophage number and activation in the Rd1 retina using well characterised antibodies to Iba1 and CD68. Iba1 is a calcium-binding protein expressed by quiescent and activated microglia as well as by infiltrating macrophages, whilst CD68 is a glycoprotein expressed only by activated, phagocytic microglia and macrophages.

**Fig. 9:**
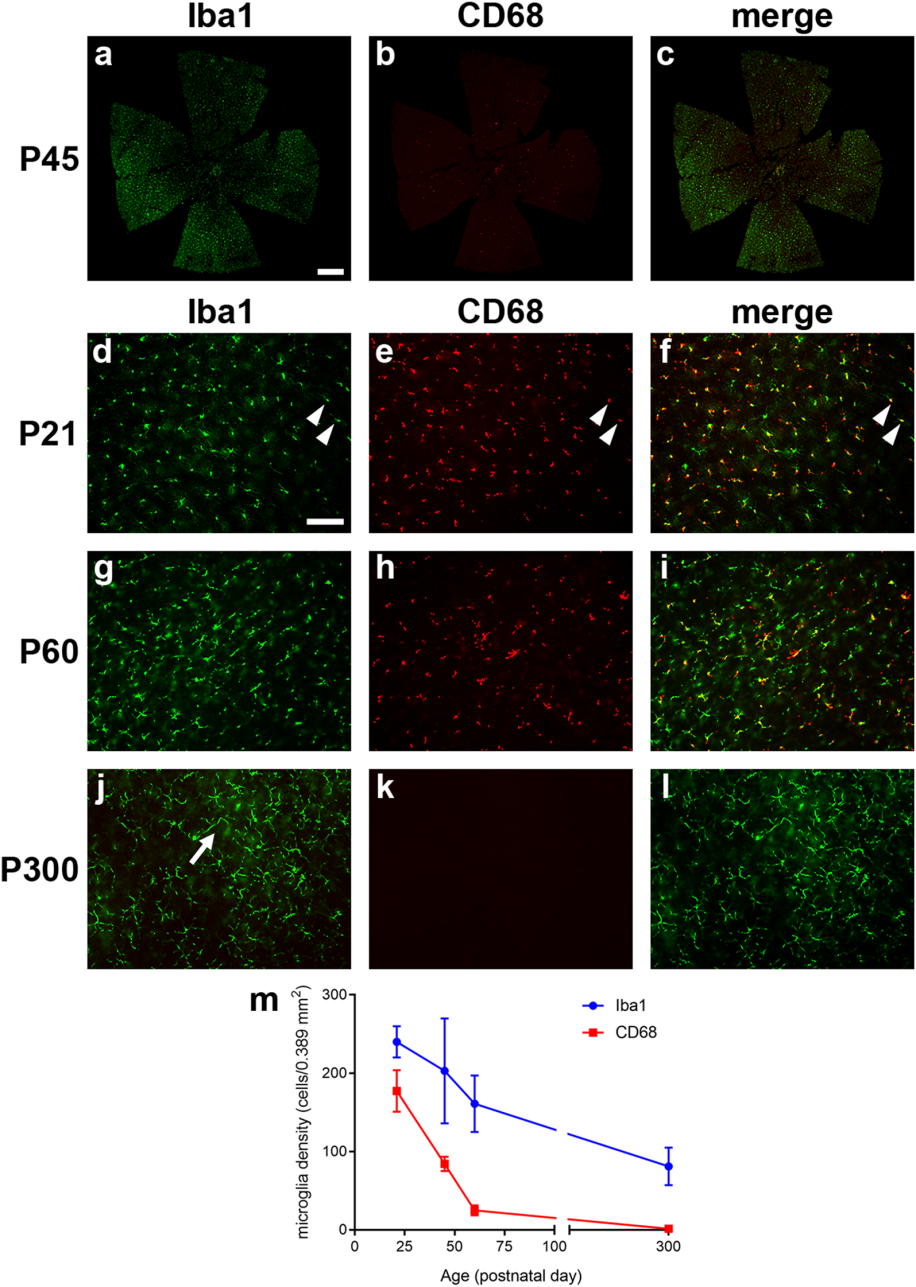
Characterisation of microglial changes in retinal wholemounts of Rd1 mice from postnatal day (P) 21 to P300. **a**–**c** Representative, low magnification, images of retinal wholemounts from P45 mice immunolabeled for CD68 (red) and Iba1 (green). **d**–**l** Representative, higher magnification, images of Rd1 retinas from P21, P60 and P300 immunolabeled for CD68 (red) and Iba1 (green). The morphology of Iba1-positive microglia changes from an activated amoeboid shape with retracted processes at P21 (arrowheads) to a quiescent appearance with ramified processes by P300 (arrow). Scale bars: **a**–**c** 500 μm; **d**–**l** 100 μm. **m** Quantification of the number of Iba1-positive and CD68-positive cells over time. Data represent mean ± SEM

We found the number of Iba1+ and CD68+ microglia/macrophages in wholemount Rd1 retinas decreased gradually from P21 to P300 (Fig. 9). The majority (79.1%) of cells at P21 were active and expressed both CD68 and Iba1. These activated microglia/macrophages presented a typical amoeboid shape with scarce dendrites. By P60, 67% of Iba1+ microglia were still present in the retina and only 17.6% expressed CD68. The inactive microglia displayed typical quiescent morphology with long dendritic processes. By P300, only 33.9% of Iba1+ microglia remained and only 2.6% were active and expressed CD68. Therefore, the activation profile of microglia/influx of macrophages paralleled the degeneration of S- and M/L-cones in the Rd1 retina, with a high rate of phagocytic activity occurring at the peak period of cone degeneration.

**Fig. 10:**
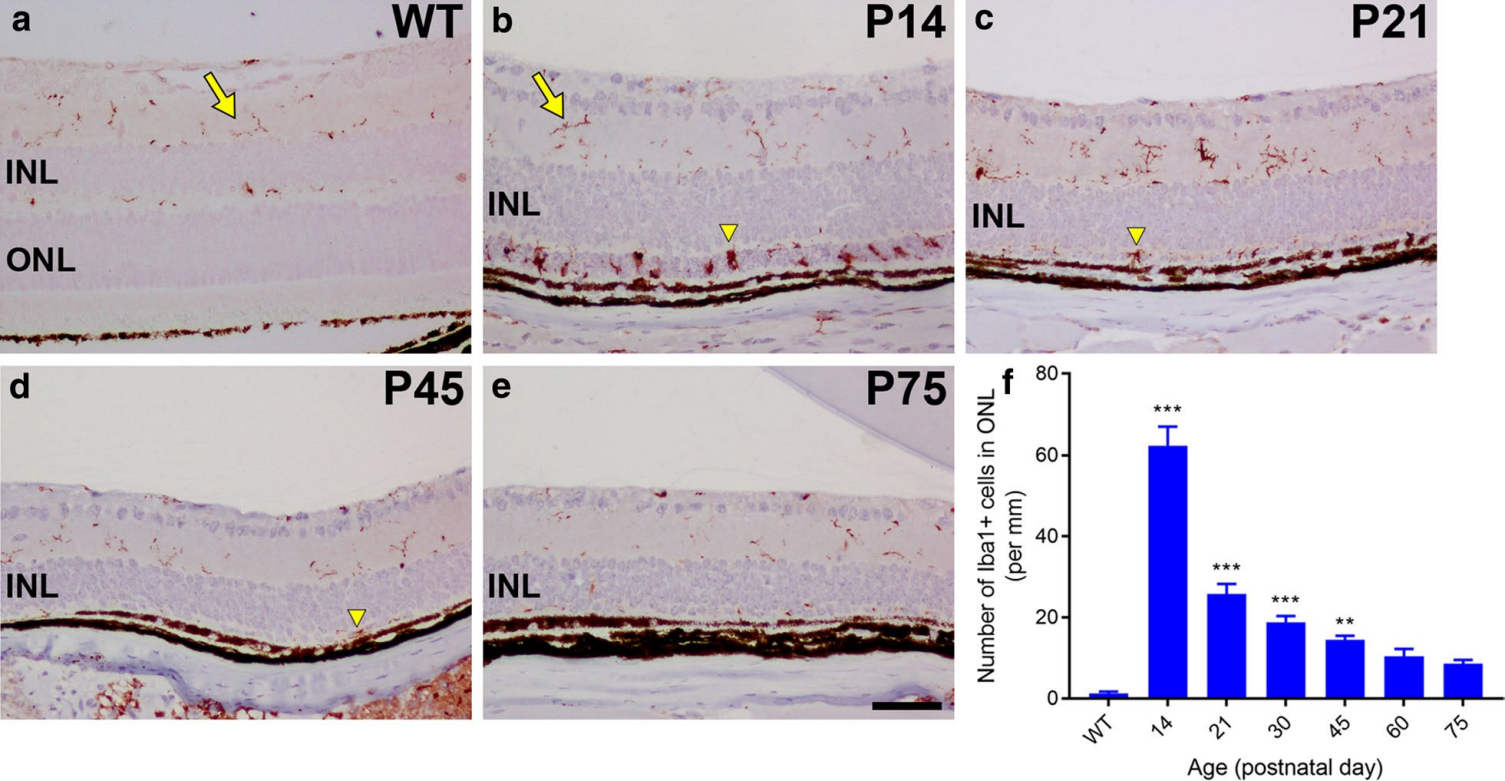
Characterisation of microglial changes in transverse retinal sections of Rd1 mice. **a**–**e** Representative images of Iba1 immunolabeling in C57BL/6 wild-type (WT) mice and in Rd1 mice from postnatal day (P) 14 to P75. In WT retinas, Iba-positive microglia are restricted to the nerve fiber, inner plexiform, and outer plexiform layers and had ramified cell processes (**a**, arrow). In Rd1 mice, Iba1-positive microglia within the inner retina retain their ramified appearance (**b**, arrow), but there is an accumulation of microglia with an amoeboid appearance (arrowhead) in the outer nuclear layer (ONL) at P14, which coincides with photoreceptor degeneration (**b**). By P21, the density of Iba1 microglia in the ONL has decreased substantially (**c**, arrowhead) and continues to decrease at later time points (**d**, **e**, arrowhead). **f** Quantification of the number of Iba1-positive cells in the ONL over time. Scale bar = 50 μm. *GCL* ganglion cell layer, *INL* inner nuclear layer

In transverse sections of the Rd1 retina (Fig. 10), a large population of Iba1+ microglia/macrophages (62 cells/mm) was present in the ONL and inner/outer segments at P14 where photoreceptor degeneration occurs. These outer retinal cells were characterised by an activated amoeboid shape, whereas the resident population of Iba+ microglia in the inner retina demonstrated a quiescent (ramified) morphology. The population of Iba1+ cells in the outer retina was greatly diminished at P21 (26 cells/mm) and decreased gradually thereafter, with very few Iba1+ cells remaining in the outer retina by P75 (9 cells/mm).

## Discussion

The results of the present study provide valuable insights into the spatio-temporal patterns of cone outer segment and cell body degeneration in Rd1 mice, substantiating and extending conclusions drawn from earlier studies. The first finding from our study is that the outer segments of S- and M/L-cones degenerate far more rapidly than their somas. Loss of both S- and M/L-opsin^+^ outer segments was fundamentally complete by P21 in the central retina, and 90% complete by P45 in the peripheral retina. In comparison, degeneration of S- and M/L-opsin^+^ cell bodies proceeded at a much slower rate. The data support the largely qualitative observations of Lin et al. [8], who observed the same temporal pattern of S- and M/L-opsin^+^ segment vs somal degeneration. The findings are also in agreement with studies that have employed the lectin peanut agglutinin—which specifically labels cone matrix sheaths—to quantify cone loss and survival in Rd1 mice. Thus, Punzo et al. [9], showed a profound decrease in cone outer segment length within the central retina by P21 and a gradual reduction in outer segment length in the peripheral retina, whilst Komeima et al. [10] documented less than 25% survival of PNA-labeled cones in all four quadrants of the retina by P35. The early loss of cone outer segments explains the profound reduction in cone function that has been measured at P21 [22].

The second quantitative finding from our study is that cone degeneration in Rd1 mice occurs far more rapidly in the central retina than in the peripheral retina. This was consistent for both S- and M/L-opsin^+^ cones and was applicable both to outer segments as well as to cell bodies. At P60, for example, survival of S- and M/L-opsin^+^ cones somas was approximately threefold greater in peripheral regions than in the central retina. The phenomenon of cone loss advancing from the central to the peripheral retina was first noted by Carter-Dawson et al. [11] and has been corroborated in a number of subsequent studies using wholemounts and transverse sections [8, 9, 12–14].

In Rd1 mice, degeneration of cone outer segments results in redistribution of opsins to the surviving cell body [8, 16, 17]. This process facilitates evaluation of the timing of somal death, with the assumption that loss of opsins from the cell body equates to actual cell demise. With regard to the spatio-temporal degeneration of cone cell bodies, the most striking feature was the marked hemispheric asymmetry in the rate of S-opsin^+^ and M/L-opsin^+^ cell body degeneration. Thus, M/L-opsin^+^ cones were more resilient to degeneration in the superior compared to the inferior retina, whilst S-opsin^+^ cones were remarkably well preserved in the inferior retina relative to the superior retina. The degeneration of both cone types in the nasal and temporal quadrants of the retina followed similar kinetics. The present data confirm and extend the earlier findings of Lin et al. [8], although some relatively minor differences were apparent, particularly with regard to S-opsin^+^ cones. Our data show that at P90 approximately one-third of S-opsin^+^ cones survived in the superior peripheral retina and 50% in the inferior peripheral retina, whereas Lin et al. noted only a small number of S-opsin^+^ cones in the far periphery of the inferior retina at this time point. The explanation may lie in the sensitivity of the respective immunohistochemical assays, or perhaps more likely with variability between different Rd1 populations. Highly significant inter-animal variability has indeed been reported in the Rd1 strain in terms of cone survival [8, 13].

The quantitative data presented in this study will prove useful for researchers that employ the Rd1 model to test potential therapeutic strategies to delay cone loss. In the majority of these studies cone quantification is the primary outcome, which is typically measured using peanut agglutinin or cone opsins. The marked difference in survival between cell bodies and outer segments is a highly relevant factor when considering both the timing of neuroprotective therapy onset and the experimental duration. The use of peanut agglutinin requires both early initiation and short experimental duration, as exemplified by Komeima et al. [10] in their investigation into the efficacy of antioxidants as neuroprotectants. If a therapy were to be initiated beyond P21, any effect on cone outer segment quantification will likely be confounded by the low baseline number of outer segments. Since cone cell bodies survive considerably longer than outer segments, quantification of somal number using antibodies to cone opsins presents a much longer time window to measuring any positive influence of a therapy. Nevertheless, the divergence between cone survival in the central and peripheral regions of the retina, as well as the topographical differences between survival of S-opsin^+^ and M/L-opsin^+^ cones necessitates careful consideration of both the experimental endpoint and the areas to be sampled. Automated, pan-retinal approaches to quantification, such as those devised by Aguado-Barriuso et al. [15, 19, 23] and Clerin et al. [14] are ideally suited to quantification of intensely labeled, discrete outer segments. Cone cell bodies, however, being larger than outer segments, less uniformly shaped, and labeling with both lower, and variable, intensity fluorescence do present a greater technical challenge. Well-considered, semi-automated approaches to counting of cone cell bodies have been devised, as for example by Punzo et al. [24, 25].

In addition to histologic methods, reliable and sensitive assessment of cone survival can be achieved by measurement of the total retinal content of mRNAs encoding cone-specific markers. Our data showed that the levels of both S-opsin and M/L-opsin mRNAs decreased in a relatively linear fashion from P14 until P90, at which point each mRNA was 16% of the WT level. Earlier work investigating S-opsin mRNA expression in Rd1 retinas found a similar rate of loss [9]. These data indicate that cones continue to synthesize opsin proteins while their outer segments degenerate, and further, that cone opsin mRNA expression is a useful, adjunct quantitative tool to measure cone injury which is not dependent upon careful image sampling nor is restricted to a narrow time window.

In the second part of this study, we describe, for the first time, the proportion and distribution of dual cones in the Rd1 retina at the onset of cone degeneration, P14, and at the approximate midpoint of cone degeneration, P60. Our data show that at P14, the superior retina comprised a minority of genuine S-cones with a similar much greater complement of genuine M/L-opsin cones and dual cones, whilst the other three retinal quadrants had broadly similar numbers of genuine S-cones, genuine M/L-cones and dual cones. This distribution is not dissimilar to the complement of the cone types in the WT mouse retina, in which 26% of cones are genuine M/L-cones, 34% are genuine S-cones, and the remaining 40% are dual cones [15]. By P60, the relative proportion of genuine S-cones in the superior peripheral retina was negligible, yet approximately 50% of surviving cones were dual cones, which accounts for the noteworthy S-opsin^+^ cone survival in this quadrant discussed earlier. In contrast, the inferior peripheral retina at P60 contained almost exclusively genuine S-cones with a tiny minority of dual cones, and no identifiable genuine M/L-cones. The remarkable preservation of genuine S-cones in the inferior peripheral retina at P60 accounts for why there was approximately threefold greater overall cone survival here than in the other three quadrants. This finding supports the previous observation by LaVail et al. [13] that there is substantially greater cone survival in the inferior vs superior retina at P60. As in the superior peripheral retina, approximately 50% of surviving cones in the nasal and temporal quadrants were dual cones. Overall, our data reveal that dual cones comprised a large percentage of surviving cones in the P60 Rd1 retina and were slightly more resilient to degeneration relative to genuine S-cones and M/L-cones in all quadrants except for the inferior retina. Understanding how dual cones survive compared to genuine S-cone and M/L-cones could help provide valuable information about the mechanisms of cone degeneration in the Rd1 mouse.

Finally, we examined the kinetics of microglial/macrophage activity during the period of cone degeneration in the Rd1 retina. Microglia are the resident immunocompetent cells of the CNS and microglial activation is recognized as the hallmark of neuroinflammation with typical morphological changes and expression of surface markers [26]. Previous studies have reported microglial activation in the Rd1 retina [27, 28], notably the comprehensive study of Zhou et al. [29], but the focus of attention has mostly been on the period during, and immediately following, rod degeneration. Less information exists describing the relationship between microglial/macrophage activity and cone degeneration. Herein, we found that the number of microglia/macrophages in the Rd1 retina decreased from P21 to P300, and, the relative proportion of activated microglia/macrophages decreased at a faster rate. The overall kinetics of microglial activity mirrored that of cone degeneration. Microglial/macrophage activity was clearly stimulated in the early period of rod breakdown and remained relatively high between P21 and P45—during the period of cone outer segment degeneration and loss of cone somas in the central retina—and low between P60 and P300—a period coincident with slow degeneration of cone cell bodies in the peripheral retina. Our data suggests a close relationship between microglia/macrophages and cone degeneration, but whether such activity is causative or occurs secondary to cone degeneration is an interesting and unresolved question.

## Conclusions

In summary, we have compared the rates of S- and M/L-opsin^+^ cell body and outer segment degeneration, described the distribution and proportion of dual cones, and examined the kinetics of microglial activation during the period of cone degeneration in the Rd1 retina. The overall results provide valuable baseline data for future studies investigating cone neuroprotection in the Rd1 mouse and, furthermore, may provide some insight into potential mechanisms of cone loss.

## Supplementary information

**Additional file 1.** Representative images of M/L-opsin^+^ and S-opsin^+^ cones in transverse retinal sections of wild-type mice. Arrows highlight outer segments. Arrowhead highlights somatic labeling, which was more prominent in S-opsin^+^ cones. Scale bar: 20 μm. INL, inner nuclear layer; ONL, outer nuclear layer.

**Additional file 2.** Visualisation of M/L-opsin^+^ and S-opsin^+^ cone segments and cell bodies in retinal wholemounts. (A) Representative high magnification images of M/L-opsin^+^ and S-opsin^+^ cones in retinal wholemounts of Rd1 mice from postnatal day 60 (B). Outer segments are identified by high fluorescent intensity (white arrows). In comparison, cell bodies label with lower fluorescent intensity (white arrowheads). These different cellular structures can, therefore, be analysed and quantified separately. (B) Image thresholding of M/L-opsin^+^ cone segments. The left image shows M/L-opsin^+^ cones in the superior peripheral quadrant of a retinal wholemount. Insets 1 and 2 are magnified views of two regions from the photomicrograph. The right panel shows the ML-opsin^+^ image overlaid with the mask derived from image thresholding to isolate outer segments (white represents areas to be quantified). It can be seen that the mask recapitulates the distribution of immunolabeled segments. Scale bar A = 50 μm; B= 100 μm.

**Additional file 3.** Representative images of rhodopsin^+^-rods in transverse sections of the Rd1 mouse central retina from postnatal day (P) 14 to P21. At P14, the outer nuclear layer is reduced to 3–4 cells in thickness. By P21, rod degeneration is almost complete. Scale bar 50 μm.

**Additional file 4.** Representative images of M/L-opsin^+^-cones in transverse sections of the Rd1 mouse mid-retina from postnatal day (P) 14 to P60. At P14, outer segments are typically swollen and misshapen, while ectopic redistribution of M/L-opsin to the cell body is frequently evident. By P21, outer nuclear layer thinning is very advanced, and M/L-opsin^+^ outer segment degeneration is considerable. M/L-opsin cell body degeneration progresses gradually from P21 to P60. Scale bar 50 μm.

**Additional file 5.** Representative images of S-opsin^+^-cones in transverse sections of the Rd1 mouse mid-retina from postnatal day (P) 14 to P60. At P14, outer segments are typically swollen and misshapen, while ectopic redistribution of S-opsin to the cell body is uniformly evident. By P21, outer nuclear layer thinning is very advanced, and S-opsin^+^ outer segment degeneration is considerable. S-opsin cell body degeneration progresses gradually from P21 to P60. Scale bar 50 μm.

**Additional file 6.** Representative, high magnification, images of S-opsin^+^ cones, M/L-opsin^+^ cones and their merged image in retinal wholemounts of C57BL/6 wild-type mice. Images from the superior (A-C), inferior (D-F, nasal (G-I) and temporal (J-L) quadrants are shown. Double labeling immunofluorescence was performed using antibodies directed against S-opsin (red) and M/L-opsin (green). Scale bar: 100 μm.

**Additional file 7.** Representative images of genuine S-cones, genuine M/L-cones and dual cones in the inferior peripheral retina of C57/BL/6 wild-type mice. Double labeling immunofluorescence of retinal wholemounts was performed using antibodies directed against S-opsin (red) and M/L-opsin (green). (A) S-opsin^+^ cones; (B) M/L-opsin^+^ cones; (C) merged image (all cones); (D) mask of genuine S-cones, (E) mask of genuine M/L-cones (F) mask of dual cones; (G) merged image (all cones) overlaid with mask of genuine S-cones; (H) merged image (all cones) overlaid with mask of genuine M/L-cones; (I) merged image (all cones) overlaid with mask of dual cones. Scale bar: 100 μm.

**Additional file 8.** Representative images of genuine S-cones, genuine M/L-cones and dual cones in the superior peripheral retina of Rd1 mice at postnatal day 14. Double labeling immunofluorescence of retinal wholemounts was performed using antibodies directed against S-opsin (red) and M/L-opsin (green). (A) S-opsin^+^ cones; (B) M/L-opsin^+^ cones; (C) merged image (all cones); (D) mask of genuine S-cones, (E) mask of genuine M/L-cones (F) mask of dual cones; (G) merged image (all cones) overlaid with mask of genuine S-cones; (H) merged image (all cones) overlaid with mask of genuine M/L-cones; (I) merged image (all cones) overlaid with mask of dual cones. Scale bar: 100 μm.

**Additional file 9.** Representative images of genuine S-cones, genuine M/L-cones and dual cones in the inferior peripheral retina of Rd1 mice at postnatal day 14. Double labeling immunofluorescence of retinal wholemounts was performed using antibodies directed against S-opsin (red) and M/L-opsin (green). (A) S-opsin^+^ cones; (B) M/L-opsin^+^ cones; (C) merged image (all cones); (D) mask of genuine S-cones, (E) mask of genuine M/L-cones (F) mask of dual cones; (G) merged image (all cones) overlaid with mask of genuine S-cones; (H) merged image (all cones) overlaid with mask of genuine M/L-cones; (I) merged image (all cones) overlaid with mask of dual cones. Scale bar: 100 μm.

**Additional file 10.** Representative images of genuine S-cones, genuine M/L-cones and dual cones in the nasal peripheral retina of Rd1 mice at postnatal day 14. Double labeling immunofluorescence of retinal wholemounts was performed using antibodies directed against S-opsin (red) and M/L-opsin (green). (A) S-opsin^+^ cones; (B) M/L-opsin^+^ cones; (C) merged image (all cones); (D) mask of genuine S-cones, (E) mask of genuine M/L-cones (F) mask of dual cones; (G) merged image (all cones) overlaid with mask of genuine S-cones; (H) merged image (all cones) overlaid with mask of genuine M/L-cones; (I) merged image (all cones) overlaid with mask of dual cones. Scale bar: 100 μm.

**Additional file 11.** Representative images of genuine S-cones, genuine M/L-cones and dual cones in the temporal peripheral retina of Rd1 mice at postnatal day 14. Double labeling immunofluorescence of retinal wholemounts was performed using antibodies directed against S-opsin (red) and M/L-opsin (green). (A) S-opsin^+^ cones; (B) M/L-opsin^+^ cones; (C) merged image (all cones); (D) mask of genuine S-cones, (E) mask of genuine M/L-cones (F) mask of dual cones; (G) merged image (all cones) overlaid with mask of genuine S-cones; (H) merged image (all cones) overlaid with mask of genuine M/L-cones; (I) merged image (all cones) overlaid with mask of dual cones. Scale bar: 100 μm.

**Additional file 12.** Representative images of genuine S-cones, genuine M/L-cones and dual cones in the superior peripheral retina of Rd1 mice at postnatal day 60. Double labeling immunofluorescence of retinal wholemounts was performed using antibodies directed against S-opsin (red) and M/L-opsin (green). (A) S-opsin^+^ cones; (B) M/L-opsin^+^ cones; (C) merged image (all cones); (D) mask of genuine S-cones, (E) mask of genuine M/L-cones (F) mask of dual cones; (G) merged image (all cones) overlaid with mask of genuine S-cones; (H) merged image (all cones) overlaid with mask of genuine M/L-cones; (I) merged image (all cones) overlaid with mask of dual cones. Scale bar: 100 μm.

**Additional file 13.** Representative images of genuine S-cones, genuine M/L-cones and dual cones in the inferior peripheral retina of Rd1 mice at postnatal day 60. Double labeling immunofluorescence of retinal wholemounts was performed using antibodies directed against S-opsin (red) and M/L-opsin (green). (A) S-opsin^+^ cones; (B) M/L-opsin^+^ cones; (C) merged image (all cones); (D) mask of genuine S-cones, (E) mask of genuine M/L-cones (F) mask of dual cones; (G) merged image (all cones) overlaid with mask of genuine S-cones; (H) merged image (all cones) overlaid with mask of genuine M/L-cones; (I) merged image (all cones) overlaid with mask of dual cones. Scale bar: 100 μm.

**Additional file 14.** Representative images of genuine S-cones, genuine M/L-cones and dual cones in the nasal peripheral retina of Rd1 mice at postnatal day 60. Double labeling immunofluorescence of retinal wholemounts was performed using antibodies directed against S-opsin (red) and M/L-opsin (green). (A) S-opsin^+^ cones; (B) M/L-opsin^+^ cones; (C) merged image (all cones); (D) mask of genuine S-cones, (E) mask of genuine M/L-cones (F) mask of dual cones; (G) merged image (all cones) overlaid with mask of genuine S-cones; (H) merged image (all cones) overlaid with mask of genuine M/L-cones; (I) merged image (all cones) overlaid with mask of dual cones. Scale bar: 100 μm.

**Additional file 15.** Representative images of genuine S-cones, genuine M/L-cones and dual cones in the temporal peripheral retina of Rd1 mice at postnatal day 60. Double labeling immunofluorescence of retinal wholemounts was performed using antibodies directed against S-opsin (red) and M/L-opsin (green). (A) S-opsin^+^ cones; (B) M/L-opsin^+^ cones; (C) merged image (all cones); (D) mask of genuine S-cones, (E) mask of genuine M/L-cones (F) mask of dual cones; (G) merged image (all cones) overlaid with mask of genuine S-cones; (H) merged image (all cones) overlaid with mask of genuine M/L-cones; (I) merged image (all cones) overlaid with mask of dual cones. Scale bar: 100 μm.

## Data Availability

The datasets used and/or analysed during the current study are included in this published article and are available from the corresponding author on reasonable request.

## Abbreviations

GCL: ganglion cell layer
I: inferior
INL: inner nuclear layer
IPL: inner plexiform layer
N: nasal
ONL: outer nuclear layer
PBS: phosphate buffered saline
P: postnatal day
Rd1: rd/rd
RPE: retinal pigment epithelium
RP: retinitis pigmentosa
S: superior
T: temporal
WT: wild-type
