# Supplementary figures and images for: Spatio-temporal characterization of S- and M/L-cone degeneration in the Rd1 mouse model of retinitis pigmentosa
Source: BMC Neurosci. 2019 Sep 3;20:46. doi: 10.1186/s12868-019-0528-2 (PMC6720080; doi:10.1186/s12868-019-0528-2)

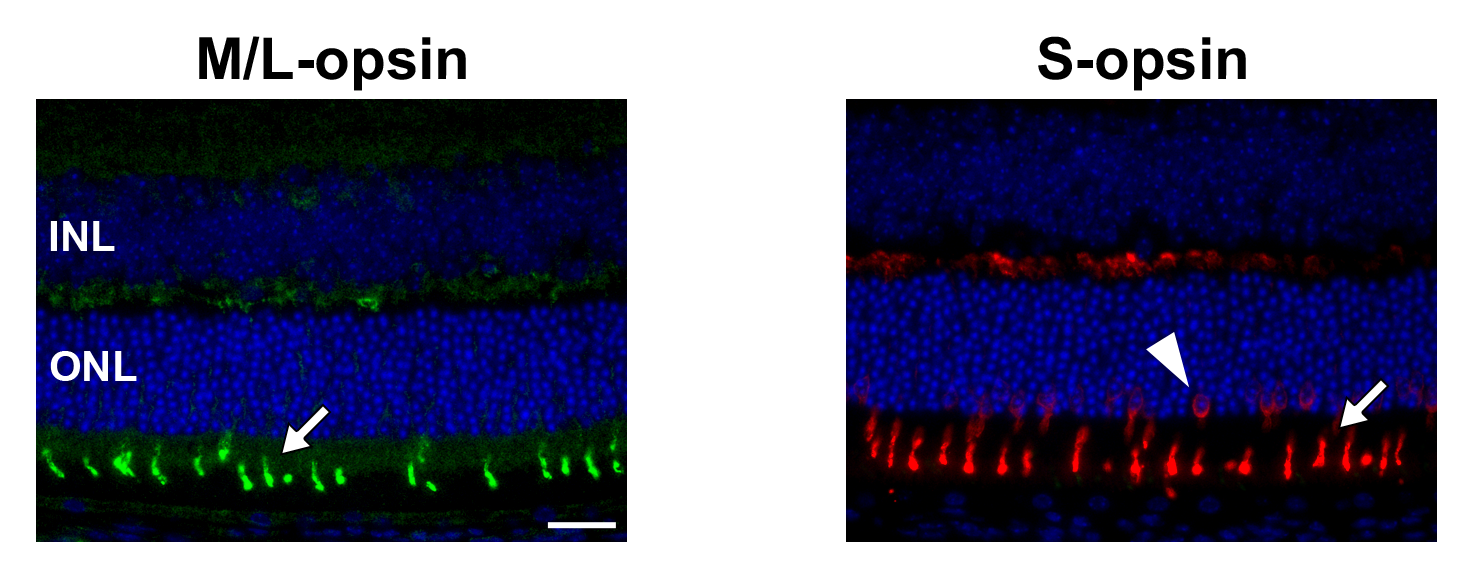

Supplement: Supplementary file 1 — Additional file 1. Representative images of M/L-opsin+ and S-opsin+ cones in transverse retinal sections of wild-type mice. Arrows highlight outer segments. Arrowhead highlights somatic labeling, which was more prominent in S-opsin+ cones. Scale bar: 20 μm. INL, inner nuclear layer; ONL, outer nuclear layer. [file 12868_2019_528_MOESM1_ESM.tif]

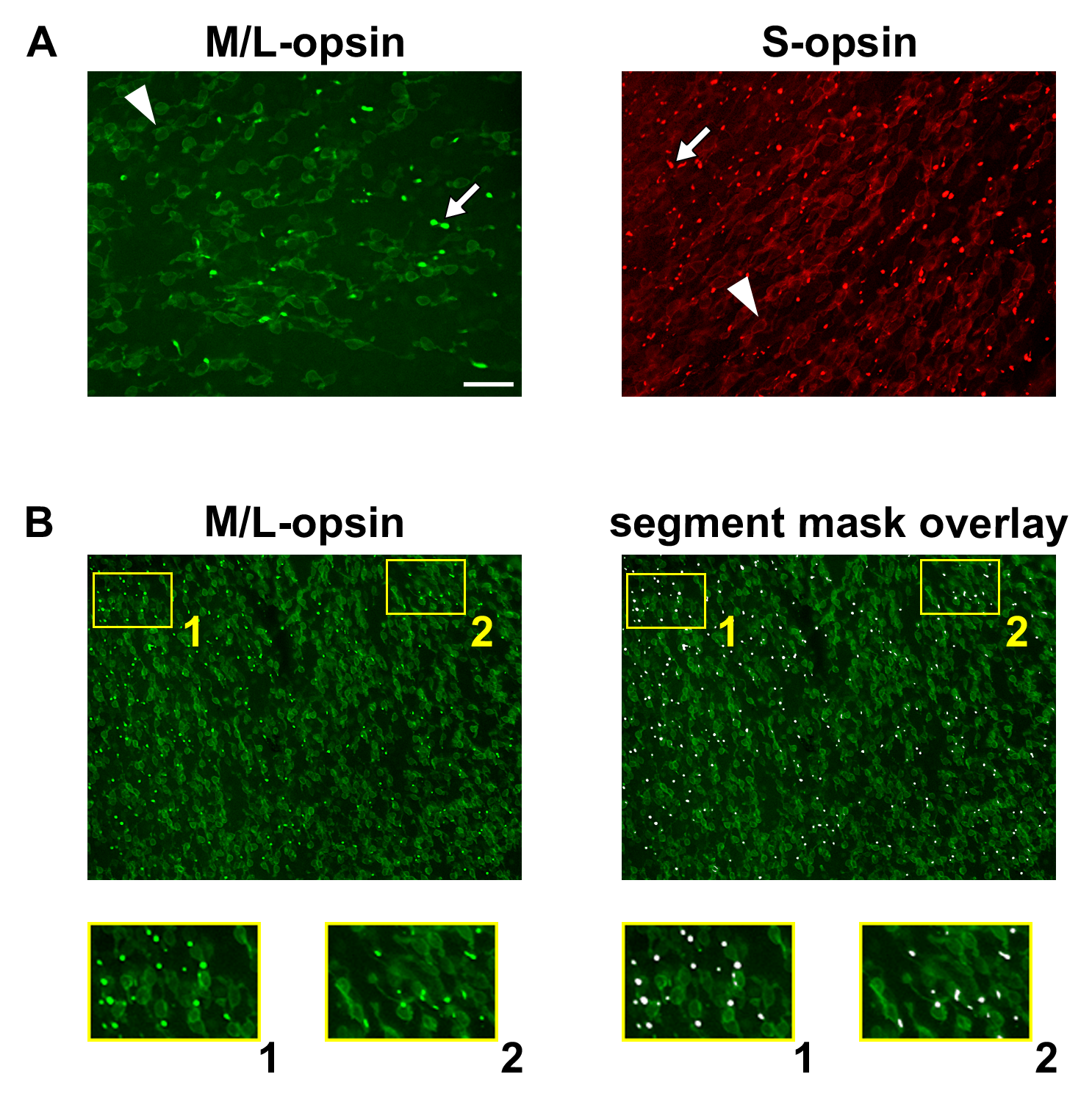

Supplement: Supplementary file 2 — Additional file 2. Visualisation of M/L-opsin+ and S-opsin+ cone segments and cell bodies in retinal wholemounts. (A) Representative high magnification images of M/L-opsin+ and S-opsin+ cones in retinal wholemounts of Rd1 mice from postnatal day 60 (B). Outer segments are identified by high fluorescent intensity (white arrows). In comparison, cell bodies label with lower fluorescent intensity (white arrowheads). These different cellular structures can, therefore, be analysed and quantified separately. (B) Image thresholding of M/L-opsin+ cone segments. The left image shows M/L-opsin+ cones in the superior peripheral quadrant of a retinal wholemount. Insets 1 and 2 are magnified views of two regions from the photomicrograph. The right panel shows the ML-opsin+ image overlaid with the mask derived from image thresholding to isolate outer segments (white represents areas to be quantified). It can be seen that the mask recapitulates the distribution of immunolabeled segments. Scale bar A = 50 μm; B= 100 μm. [file 12868_2019_528_MOESM2_ESM.tif]

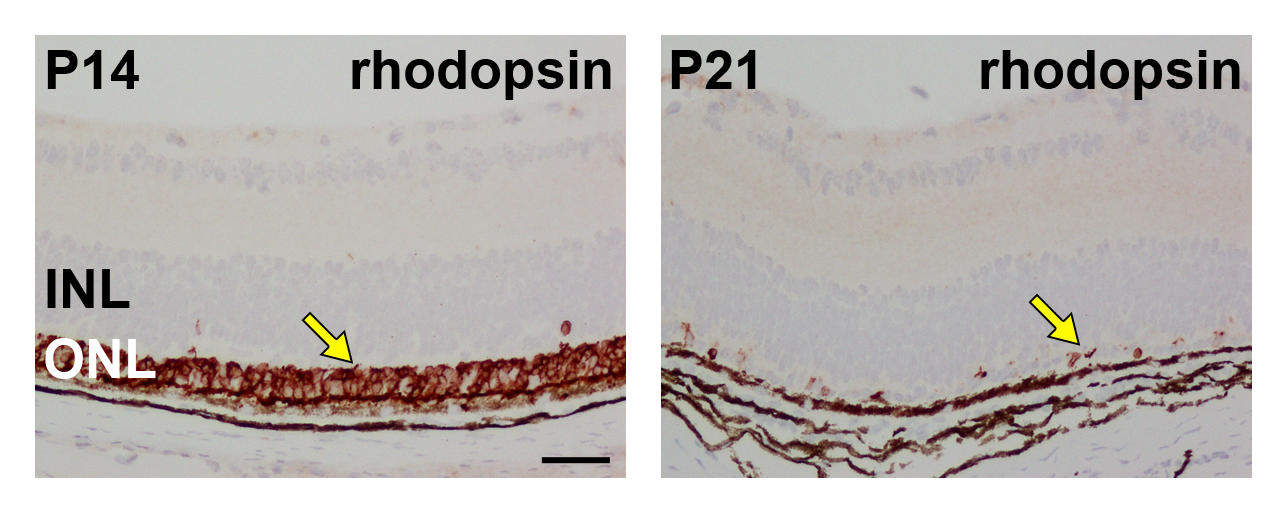

Supplement: Supplementary file 3 — Additional file 3. Representative images of rhodopsin+-rods in transverse sections of the Rd1 mouse central retina from postnatal day (P) 14 to P21. At P14, the outer nuclear layer is reduced to 3–4 cells in thickness. By P21, rod degeneration is almost complete. Scale bar 50 μm. [file 12868_2019_528_MOESM3_ESM.tif]

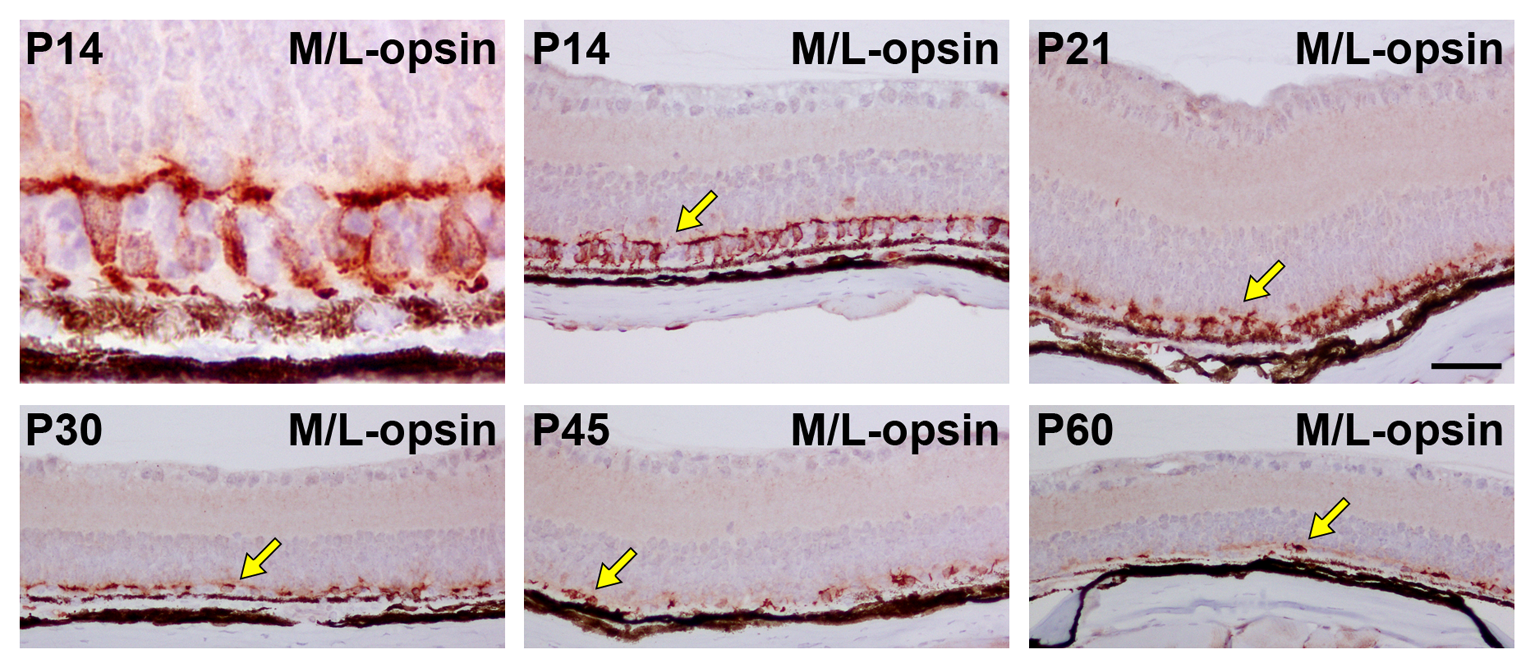

Supplement: Supplementary file 4 — Additional file 4. Representative images of M/L-opsin+-cones in transverse sections of the Rd1 mouse mid-retina from postnatal day (P) 14 to P60. At P14, outer segments are typically swollen and misshapen, while ectopic redistribution of M/L-opsin to the cell body is frequently evident. By P21, outer nuclear layer thinning is very advanced, and M/L-opsin+ outer segment degeneration is considerable. M/L-opsin cell body degeneration progresses gradually from P21 to P60. Scale bar 50 μm. [file 12868_2019_528_MOESM4_ESM.tif]

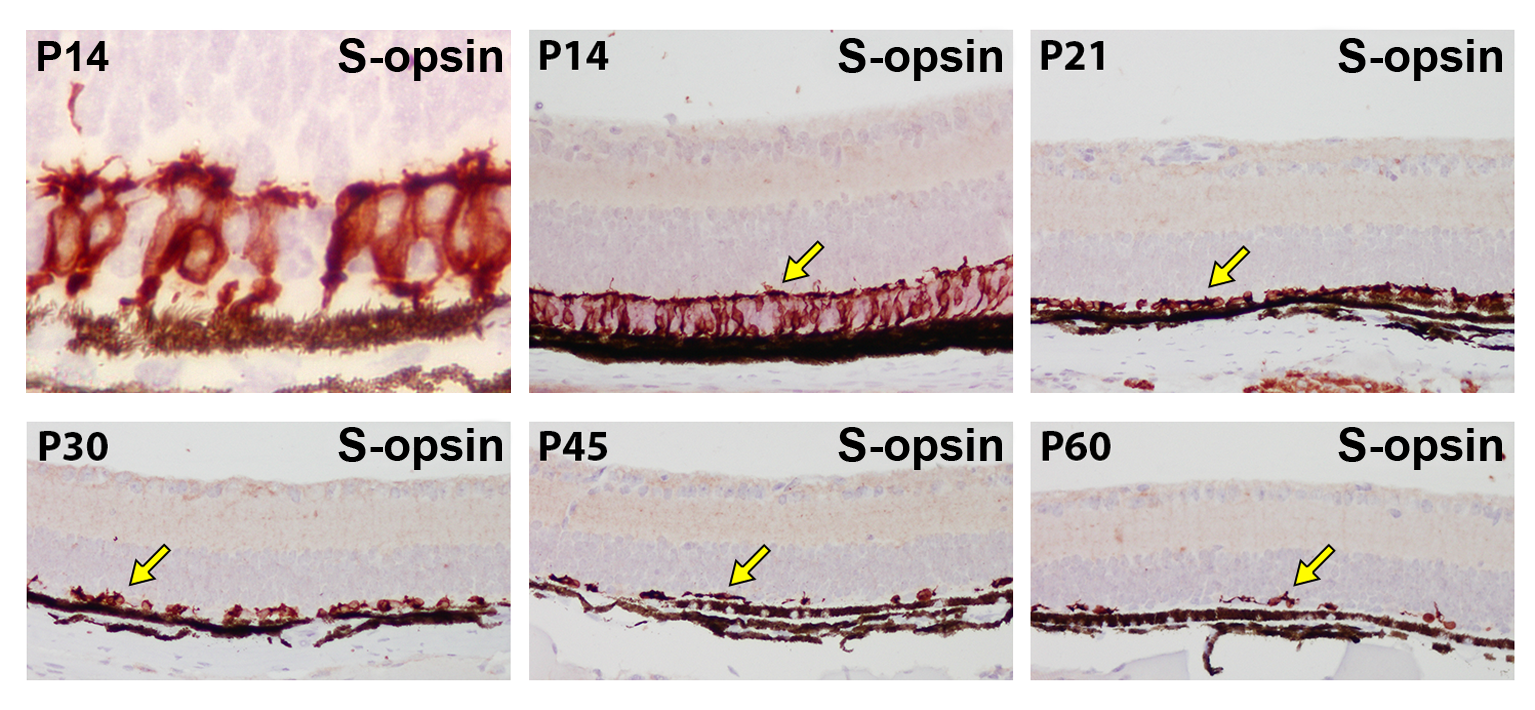

Supplement: Supplementary file 5 — Additional file 5. Representative images of S-opsin+-cones in transverse sections of the Rd1 mouse mid-retina from postnatal day (P) 14 to P60. At P14, outer segments are typically swollen and misshapen, while ectopic redistribution of S-opsin to the cell body is uniformly evident. By P21, outer nuclear layer thinning is very advanced, and S-opsin+ outer segment degeneration is considerable. S-opsin cell body degeneration progresses gradually from P21 to P60. Scale bar 50 μm. [file 12868_2019_528_MOESM5_ESM.tif]

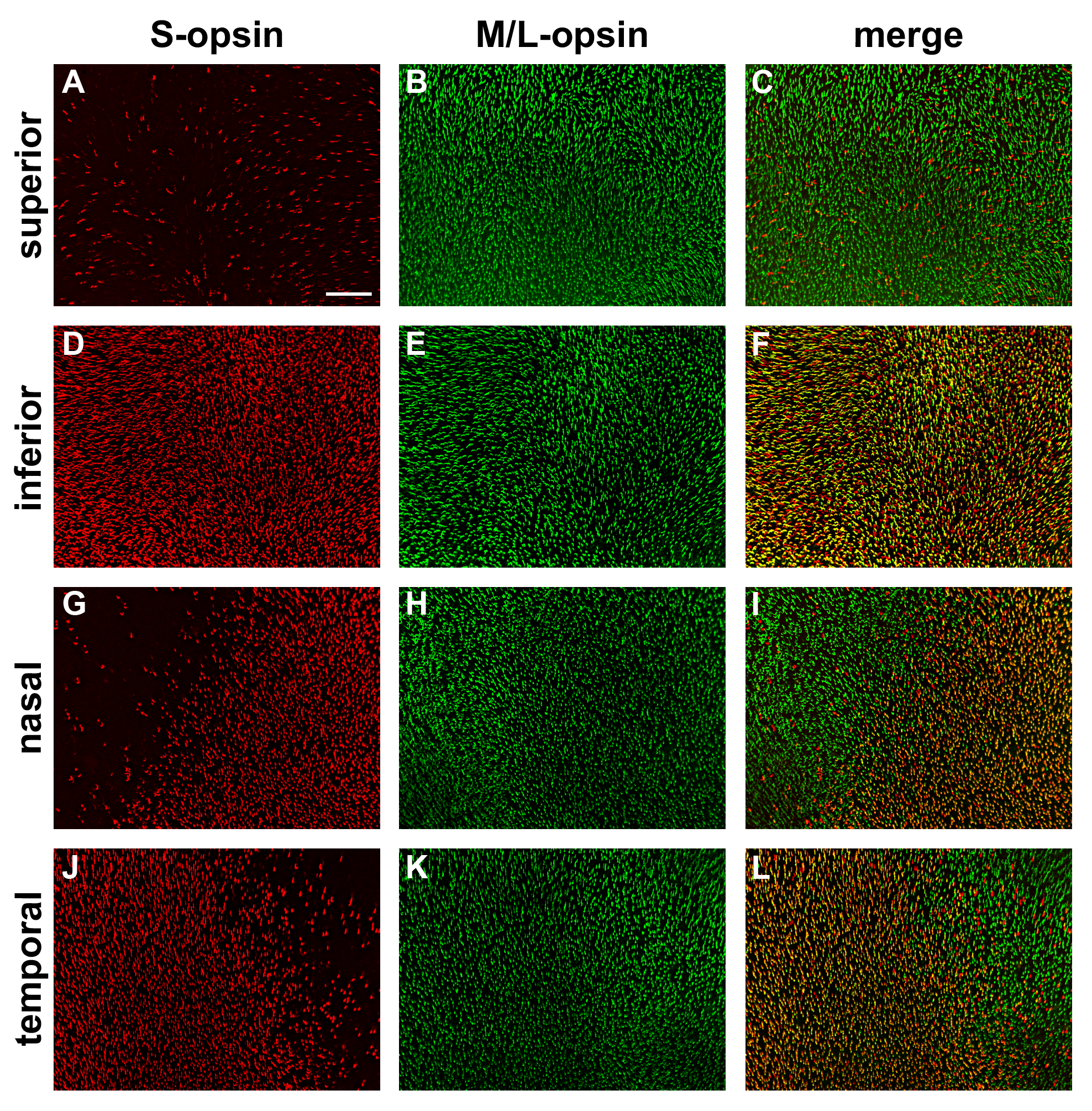

Supplement: Supplementary file 6 — Additional file 6. Representative, high magnification, images of S-opsin+ cones, M/L-opsin+ cones and their merged image in retinal wholemounts of C57BL/6 wild-type mice. Images from the superior (A-C), inferior (D-F, nasal (G-I) and temporal (J-L) quadrants are shown. Double labeling immunofluorescence was performed using antibodies directed against S-opsin (red) and M/L-opsin (green). Scale bar: 100 μm. [file 12868_2019_528_MOESM6_ESM.tif]

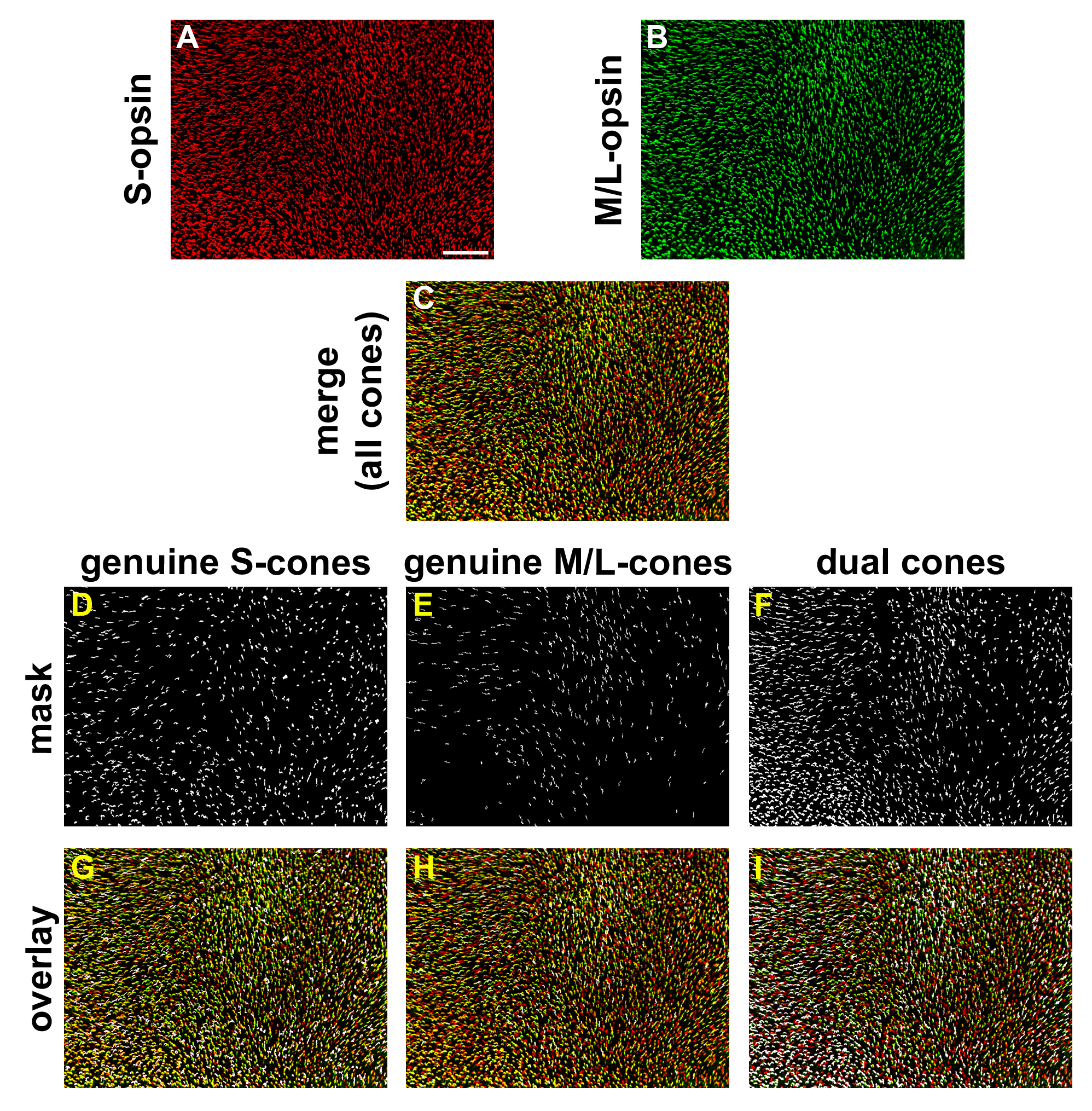

Supplement: Supplementary file 7 — Additional file 7. Representative images of genuine S-cones, genuine M/L-cones and dual cones in the inferior peripheral retina of C57/BL/6 wild-type mice. Double labeling immunofluorescence of retinal wholemounts was performed using antibodies directed against S-opsin (red) and M/L-opsin (green). (A) S-opsin+ cones; (B) M/L-opsin+ cones; (C) merged image (all cones); (D) mask of genuine S-cones, (E) mask of genuine M/L-cones (F) mask of dual cones; (G) merged image (all cones) overlaid with mask of genuine S-cones; (H) merged image (all cones) overlaid with mask of genuine M/L-cones; (I) merged image (all cones) overlaid with mask of dual cones. Scale bar: 100 μm. [file 12868_2019_528_MOESM7_ESM.tif]

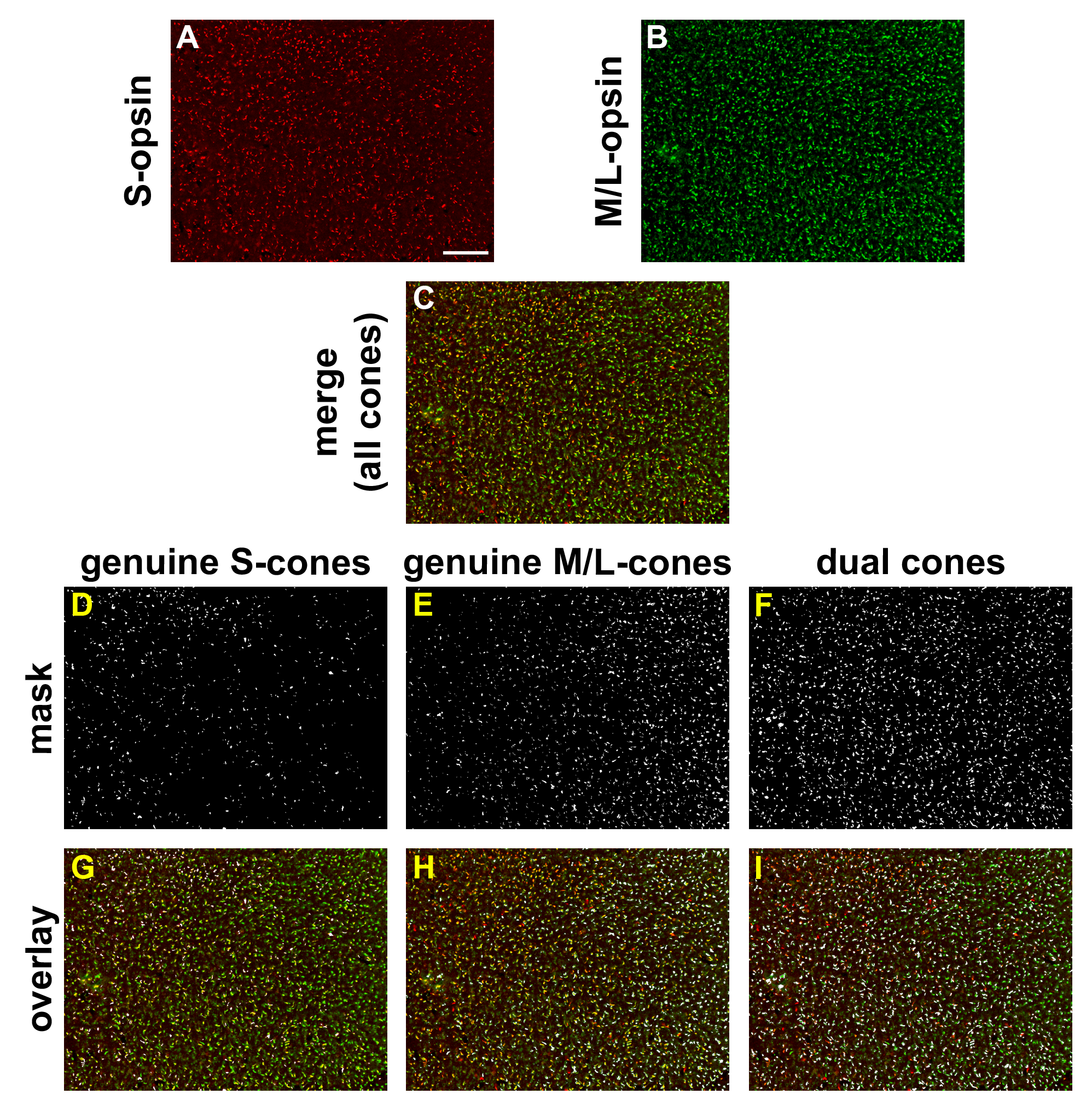

Supplement: Supplementary file 8 — Additional file 8. Representative images of genuine S-cones, genuine M/L-cones and dual cones in the superior peripheral retina of Rd1 mice at postnatal day 14. Double labeling immunofluorescence of retinal wholemounts was performed using antibodies directed against S-opsin (red) and M/L-opsin (green). (A) S-opsin+ cones; (B) M/L-opsin+ cones; (C) merged image (all cones); (D) mask of genuine S-cones, (E) mask of genuine M/L-cones (F) mask of dual cones; (G) merged image (all cones) overlaid with mask of genuine S-cones; (H) merged image (all cones) overlaid with mask of genuine M/L-cones; (I) merged image (all cones) overlaid with mask of dual cones. Scale bar: 100 μm. [file 12868_2019_528_MOESM8_ESM.tif]

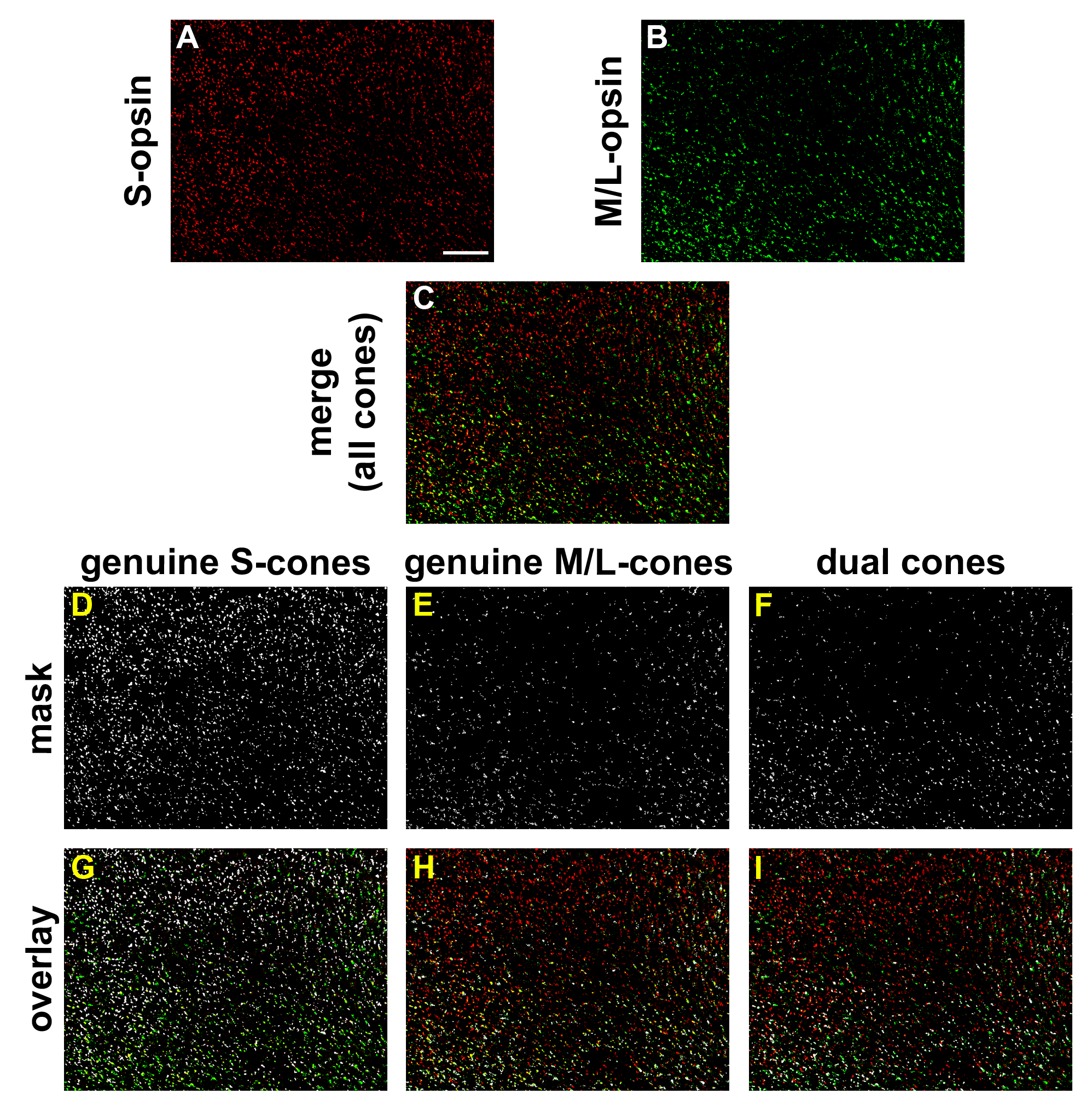

Supplement: Supplementary file 9 — Additional file 9. Representative images of genuine S-cones, genuine M/L-cones and dual cones in the inferior peripheral retina of Rd1 mice at postnatal day 14. Double labeling immunofluorescence of retinal wholemounts was performed using antibodies directed against S-opsin (red) and M/L-opsin (green). (A) S-opsin+ cones; (B) M/L-opsin+ cones; (C) merged image (all cones); (D) mask of genuine S-cones, (E) mask of genuine M/L-cones (F) mask of dual cones; (G) merged image (all cones) overlaid with mask of genuine S-cones; (H) merged image (all cones) overlaid with mask of genuine M/L-cones; (I) merged image (all cones) overlaid with mask of dual cones. Scale bar: 100 μm. [file 12868_2019_528_MOESM9_ESM.tif]

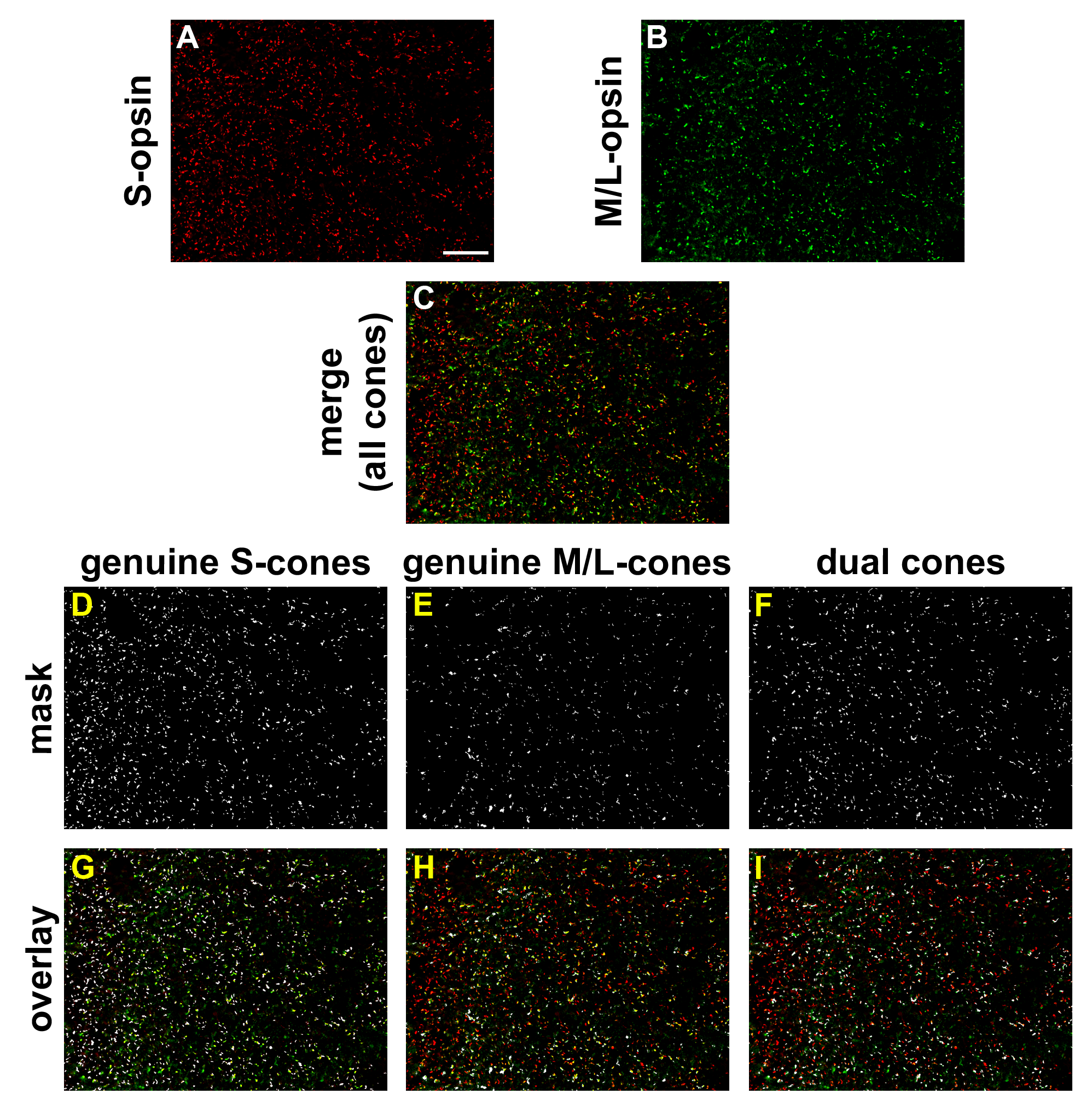

Supplement: Supplementary file 10 — Additional file 10. Representative images of genuine S-cones, genuine M/L-cones and dual cones in the nasal peripheral retina of Rd1 mice at postnatal day 14. Double labeling immunofluorescence of retinal wholemounts was performed using antibodies directed against S-opsin (red) and M/L-opsin (green). (A) S-opsin+ cones; (B) M/L-opsin+ cones; (C) merged image (all cones); (D) mask of genuine S-cones, (E) mask of genuine M/L-cones (F) mask of dual cones; (G) merged image (all cones) overlaid with mask of genuine S-cones; (H) merged image (all cones) overlaid with mask of genuine M/L-cones; (I) merged image (all cones) overlaid with mask of dual cones. Scale bar: 100 μm. [file 12868_2019_528_MOESM10_ESM.tif]

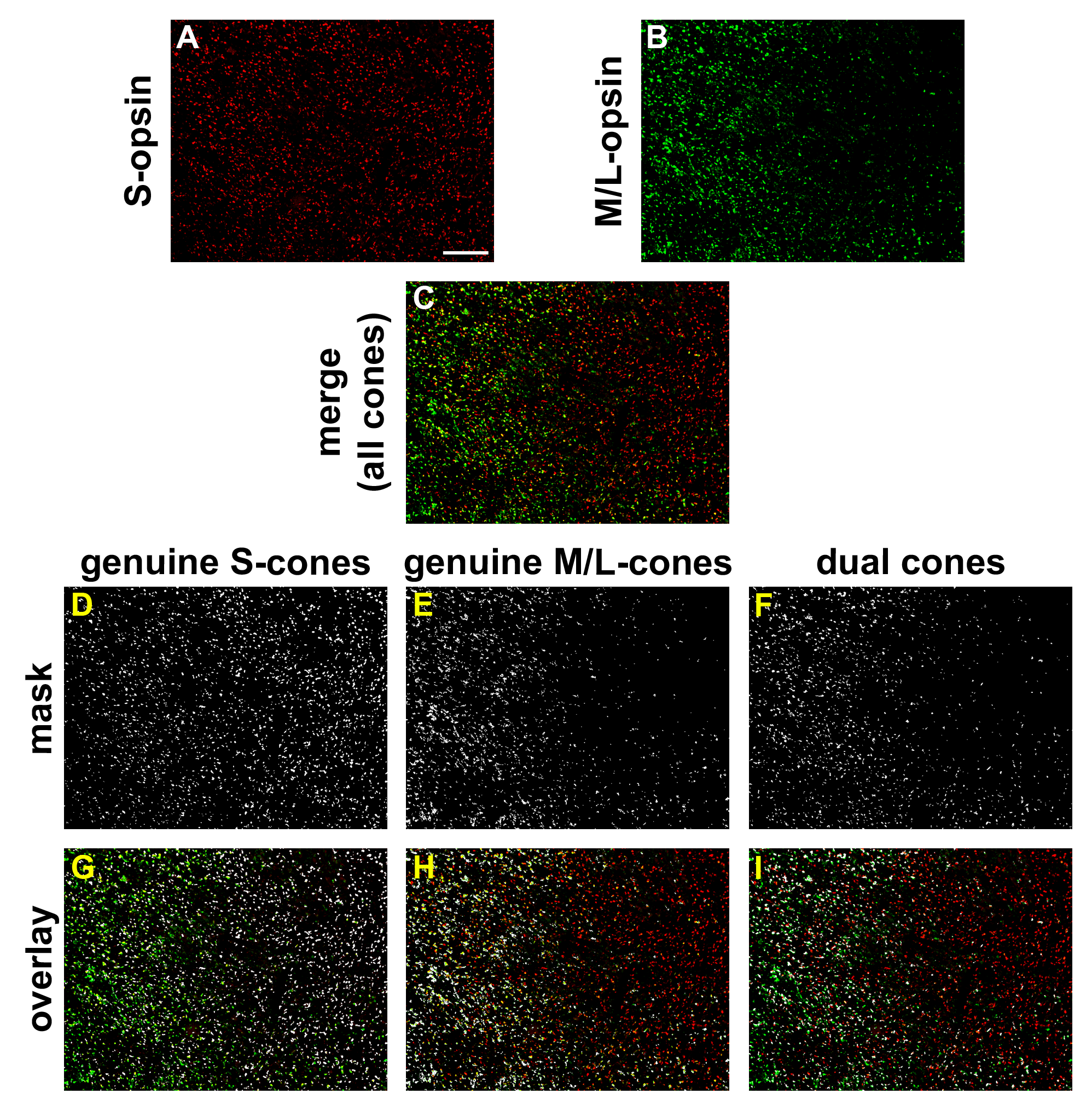

Supplement: Supplementary file 11 — Additional file 11. Representative images of genuine S-cones, genuine M/L-cones and dual cones in the temporal peripheral retina of Rd1 mice at postnatal day 14. Double labeling immunofluorescence of retinal wholemounts was performed using antibodies directed against S-opsin (red) and M/L-opsin (green). (A) S-opsin+ cones; (B) M/L-opsin+ cones; (C) merged image (all cones); (D) mask of genuine S-cones, (E) mask of genuine M/L-cones (F) mask of dual cones; (G) merged image (all cones) overlaid with mask of genuine S-cones; (H) merged image (all cones) overlaid with mask of genuine M/L-cones; (I) merged image (all cones) overlaid with mask of dual cones. Scale bar: 100 μm. [file 12868_2019_528_MOESM11_ESM.tif]

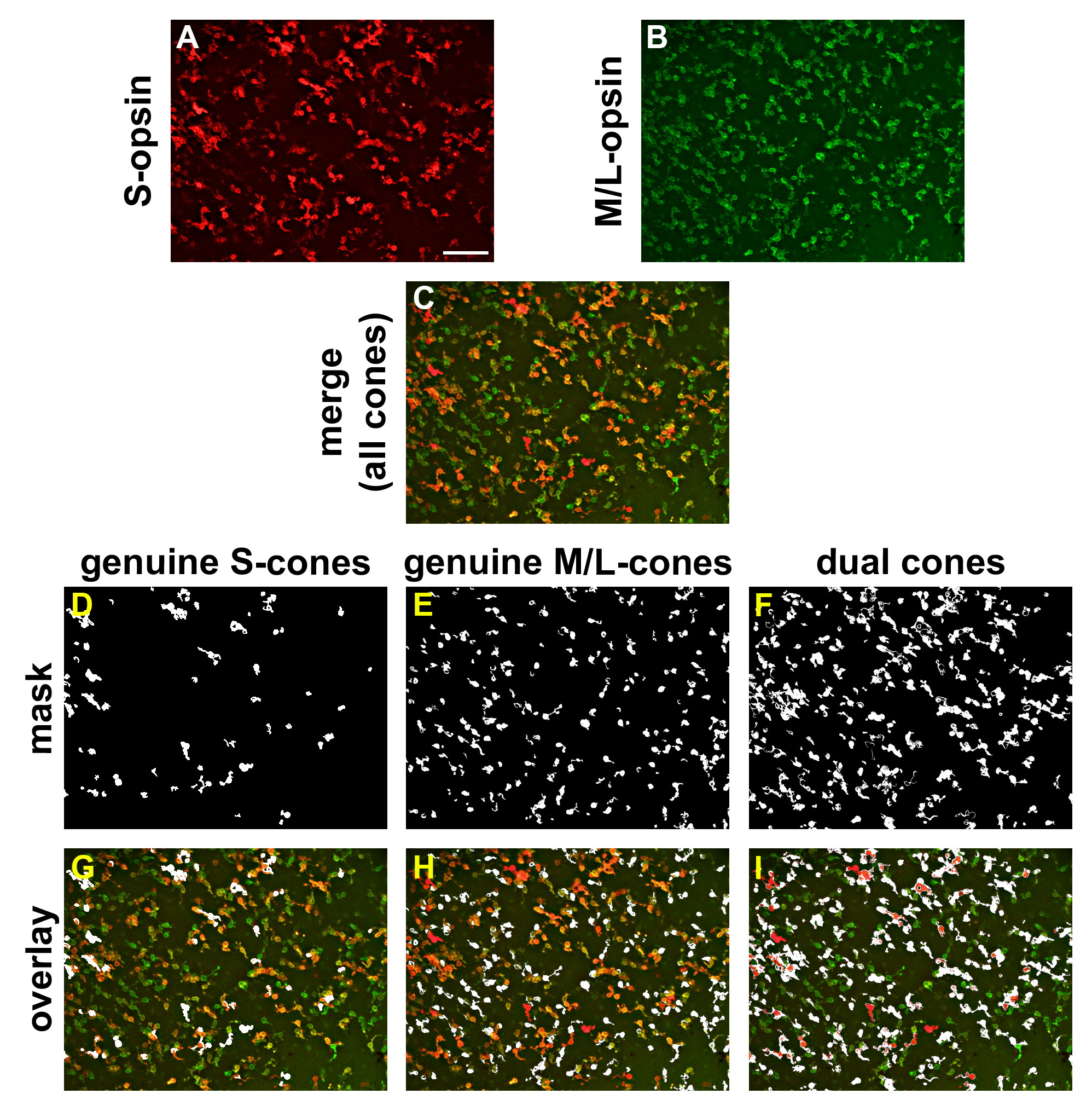

Supplement: Supplementary file 12 — Additional file 12. Representative images of genuine S-cones, genuine M/L-cones and dual cones in the superior peripheral retina of Rd1 mice at postnatal day 60. Double labeling immunofluorescence of retinal wholemounts was performed using antibodies directed against S-opsin (red) and M/L-opsin (green). (A) S-opsin+ cones; (B) M/L-opsin+ cones; (C) merged image (all cones); (D) mask of genuine S-cones, (E) mask of genuine M/L-cones (F) mask of dual cones; (G) merged image (all cones) overlaid with mask of genuine S-cones; (H) merged image (all cones) overlaid with mask of genuine M/L-cones; (I) merged image (all cones) overlaid with mask of dual cones. Scale bar: 100 μm. [file 12868_2019_528_MOESM12_ESM.tif]

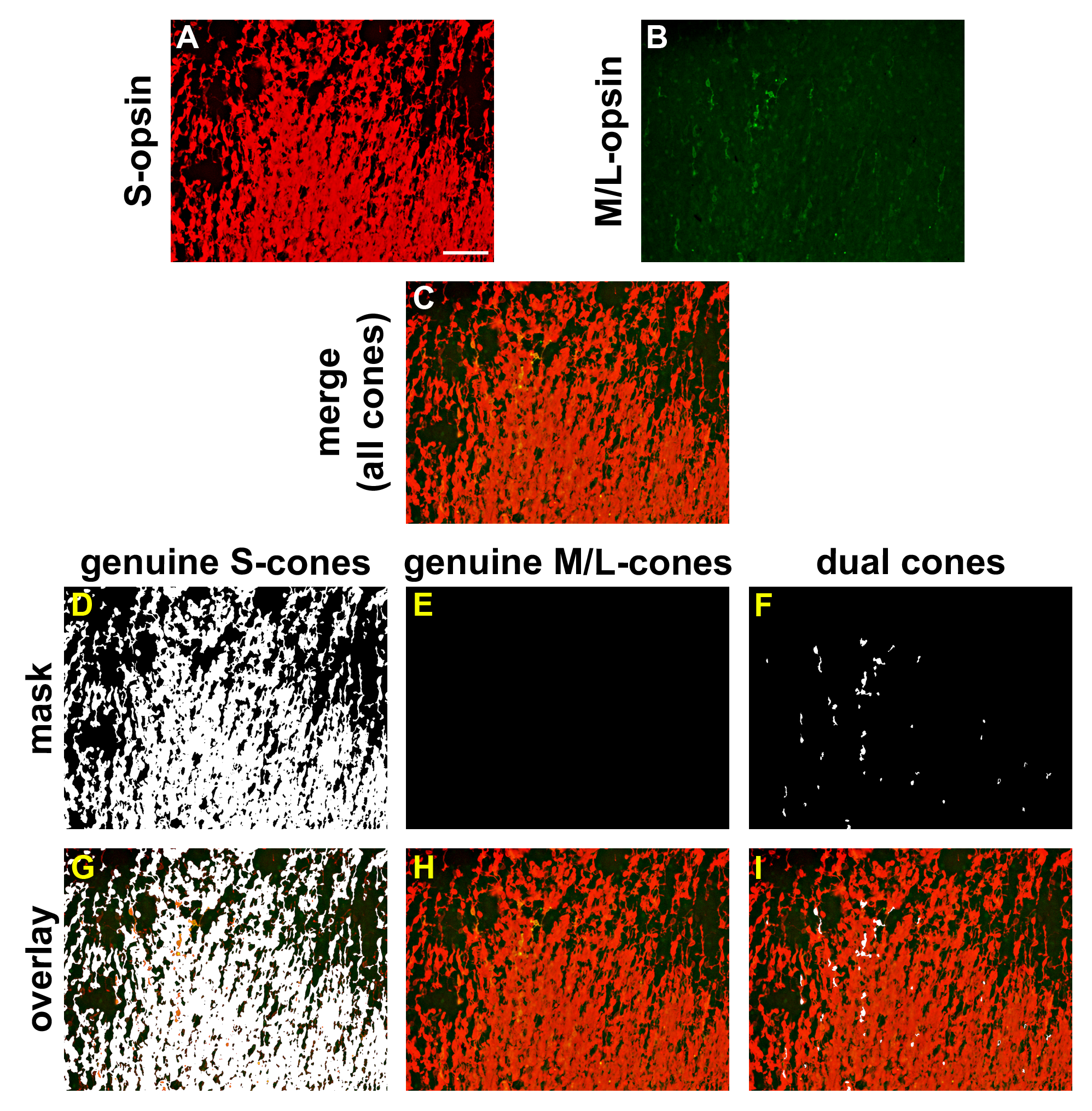

Supplement: Supplementary file 13 — Additional file 13. Representative images of genuine S-cones, genuine M/L-cones and dual cones in the inferior peripheral retina of Rd1 mice at postnatal day 60. Double labeling immunofluorescence of retinal wholemounts was performed using antibodies directed against S-opsin (red) and M/L-opsin (green). (A) S-opsin+ cones; (B) M/L-opsin+ cones; (C) merged image (all cones); (D) mask of genuine S-cones, (E) mask of genuine M/L-cones (F) mask of dual cones; (G) merged image (all cones) overlaid with mask of genuine S-cones; (H) merged image (all cones) overlaid with mask of genuine M/L-cones; (I) merged image (all cones) overlaid with mask of dual cones. Scale bar: 100 μm. [file 12868_2019_528_MOESM13_ESM.tif]

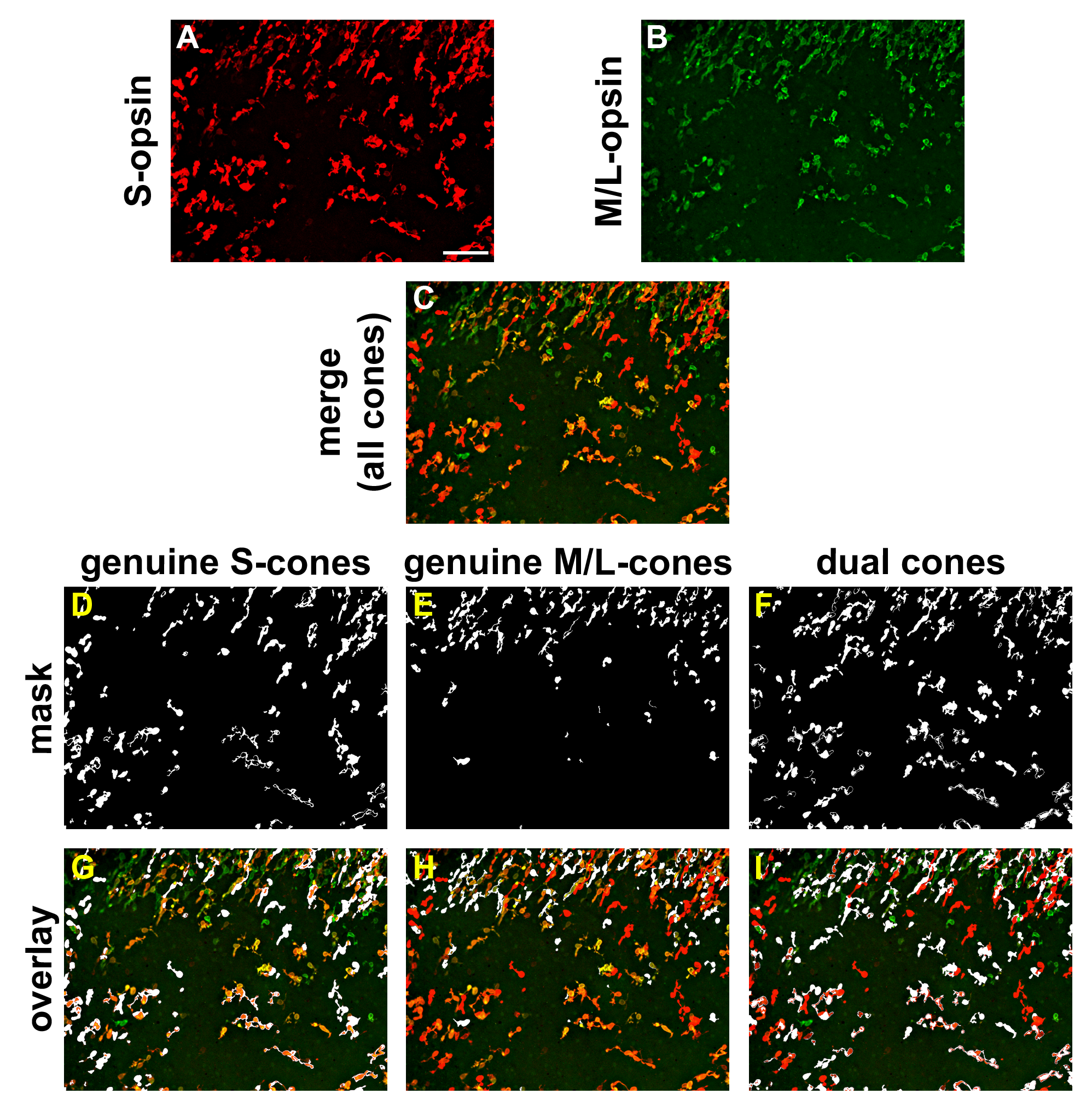

Supplement: Supplementary file 14 — Additional file 14. Representative images of genuine S-cones, genuine M/L-cones and dual cones in the nasal peripheral retina of Rd1 mice at postnatal day 60. Double labeling immunofluorescence of retinal wholemounts was performed using antibodies directed against S-opsin (red) and M/L-opsin (green). (A) S-opsin+ cones; (B) M/L-opsin+ cones; (C) merged image (all cones); (D) mask of genuine S-cones, (E) mask of genuine M/L-cones (F) mask of dual cones; (G) merged image (all cones) overlaid with mask of genuine S-cones; (H) merged image (all cones) overlaid with mask of genuine M/L-cones; (I) merged image (all cones) overlaid with mask of dual cones. Scale bar: 100 μm. [file 12868_2019_528_MOESM14_ESM.tif]

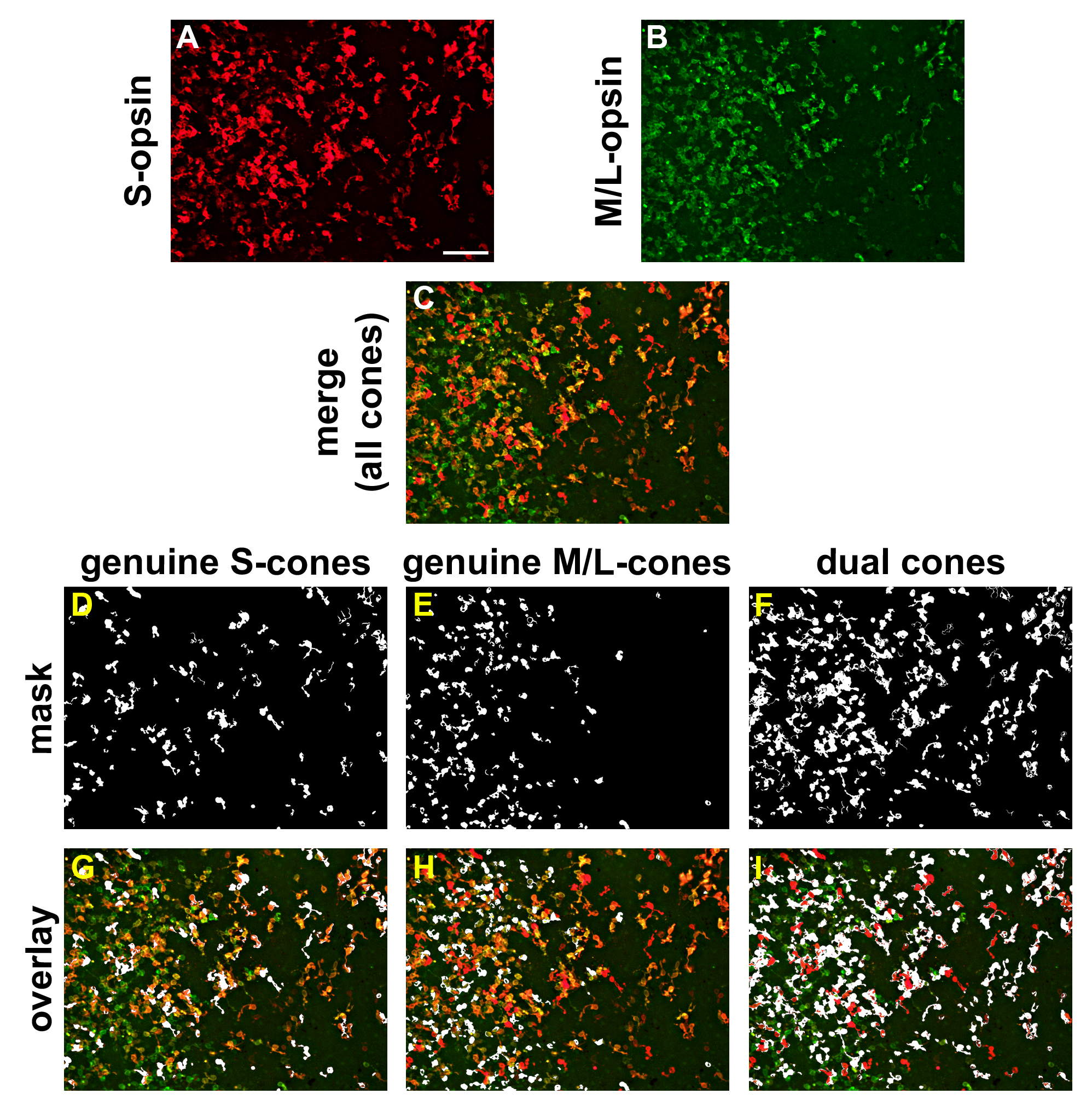

Supplement: Supplementary file 15 — Additional file 15. Representative images of genuine S-cones, genuine M/L-cones and dual cones in the temporal peripheral retina of Rd1 mice at postnatal day 60. Double labeling immunofluorescence of retinal wholemounts was performed using antibodies directed against S-opsin (red) and M/L-opsin (green). (A) S-opsin+ cones; (B) M/L-opsin+ cones; (C) merged image (all cones); (D) mask of genuine S-cones, (E) mask of genuine M/L-cones (F) mask of dual cones; (G) merged image (all cones) overlaid with mask of genuine S-cones; (H) merged image (all cones) overlaid with mask of genuine M/L-cones; (I) merged image (all cones) overlaid with mask of dual cones. Scale bar: 100 μm. [file 12868_2019_528_MOESM15_ESM.tif]
